# Cytotoxic Effects of New Palladium(II) Complexes with Thiazine or Thiazoline Derivative Ligands in Tumor Cell Lines

**DOI:** 10.3390/pharmaceutics15020696

**Published:** 2023-02-18

**Authors:** Elena Fernández-Delgado, Samuel Estirado, Ana B. Rodríguez, Francisco Luna-Giles, Emilio Viñuelas-Zahínos, Javier Espino, José Antonio Pariente

**Affiliations:** 1 Neuroimmunophysiology and Chrononutrition Research Group, Department of Physiology, Faculty of Science, University of Extremadura, 06006 Badajoz, Spain; 2 Coordination Chemistry Research Group, Department of Organic and Inorganic Chemistry, Faculty of Science, University of Extremadura, 06006 Badajoz, Spain

**Keywords:** cytotoxicity, apoptosis, Pd(II) complexes, chemotherapeutics, N,S-heterocycles

## Abstract

The synthesis of analogs of cisplatin, which is a widely used chemotherapeutic agent, using other metal centers could be an alternative for cancer treatment. Pd(II) could be a substitute for Pt(II) due to its coordination chemistry similarity. For that reason, six squared-planar Pd(II) complexes with thiazine and thiazoline ligands and formula [PdCl_2_(L)] were synthesized and characterized in this work. The potential anticarcinogenic ability of the compounds was studied via cytotoxicity assay in three different human tumor cell lines, i.e., epithelial cervix carcinoma (HeLa), promyelocytic leukemia (HL-60), and histiocytic lymphoma (U-937). Data obtained showed that complexes with methyl substitutions did not modify cell viability, while no-methyl substituted compounds had a moderate cytotoxic effect on all three cell lines. The complexes with phenyl substitutions displayed the lowest IC_50_ values, which ranged between 46.39 ± 3.99 μM and 62.74 ± 6.45 μM. Moreover, Pd accumulation inside the cell was observed after incubation with any of the four complexes mentioned, and the two complexes with phenyl rings were found to induce an increase in the percentage of apoptotic cells. These results suggested that the presence of bulky substitutions on the ligands such as phenyl groups may influence the cytotoxicity of the chemotherapeutic agents synthesized.

## 1. Introduction

Cancer remains a leading cause of mortality worldwide, and according to the World Health Organization (WHO) more than 10 million deaths were correlated with cancer in 2020, or nearly one in six [1]. Cisplatin and its derivatives are chemotherapeutic drugs frequently utilized for treating numerous types of cancers, as they can trigger cell death mechanisms in cancer cells [2]. The major drawback these drugs have is the serious side effects they produce due to their cytotoxic activity, which does not distinguish between cancerous and healthy cells [3]. To control this disease, new drugs with higher effectiveness and fewer side effects are necessary. For this reason, several recent investigations have focused their efforts on the synthesis of organometallic compounds which include transition metals and chelates [4]. In this context, palladium compounds have been explored as alternatives for platinum compounds as antitumor drugs due to their structural and chemical similarities and their coordination capability [2,4,5]. However, from a thermodynamic and kinetic perspective, Pd(II) complexes are in general more unstable than Pt(II) ones, being around 10^5^ times more reactive [6] and forming unsought chemical species before reaching their target. All these handicaps conduce to a limited anticancer activity or even inactivity and toxicity [2]. These drawbacks can be overcome by decreasing the high reactivity of the Pd(II) ion by forming more stable complexes through the using of suitable chelating agents [2,7]. Research in this field has already led to the discovery of a Pd(II) complex, padeliporfin (TOOKAD^®^), which has already been approved for clinical use [8].

A recent review on the subject indicates that the utilization of bulky ligands may help to increase the activity of Pd(II) complexes while reducing their reactivity. Nevertheless, this may also lead to a larger distortion of DNA as a consequence of palladium–DNA binding. This review also shows that in a specified series of palladium analogs, the greater lipophilicity increases the antitumor activity probably as a result of an enhancement of transport across the cellular membrane [9]. In this sense, the formation of new metal complexes with bioactive ligands containing heterocycles with N- and S-donor atoms in their structure can be a good strategy in the search for new drugs. Thus, several Pd(II) complexes containing thiazoline, thiazine, or pyrazole rings showed cytotoxic activity [10,11]. Furthermore, our previous results showed that a thiazoline-based Pd(II) complex decreases cell viability, triggers caspase-9-dependent apoptosis, and elicits oxidative DNA damage of human leukemic HL-60 cells, without affecting the viability of freshly isolated human leukocytes [12]. Similarly, a Pd(II) complex with a thiazoline derivative ligand decreased cell viability of histiocytic lymphoma U-937 cells, which was associated with caspase-3 and -9 activation and intracellular ROS production [13], whereas a Pd(II) complex with a thiazine-pyridine derivative ligand did not induce remarkable cytotoxicity in HL-60, U-937 and SK-OV 3 (ovarian adenocarcinoma) cells [14].

**Scheme 1:**
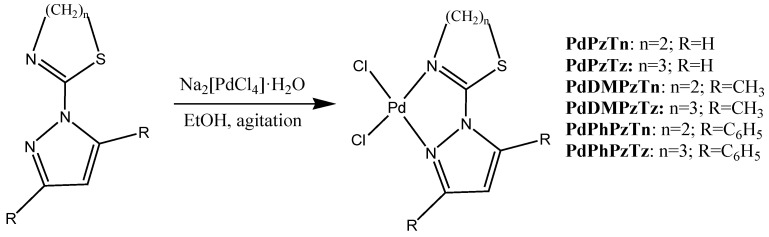
Synthesis of PdPzTn, PdPzTz, PdDMPzTn, PdDMPzTz, PdDPhPzTn and PdDPhPzTz.

Considering the relevance of the ligand structure in the complexes, our group recently published an article with some Pt(II) complexes where the presence of aromatic groups in the ligand augmented the accumulation and cellular uptake of the metallodrugs, therefore increasing the complexes’ cytotoxicity against HeLa (epithelial cervix carcinoma), HL-60 and U-937 cell lines [15]. Thus, we become interested in investigating whether also ligands with bulky and aromatic groups can enhance the cytotoxic activity of Pd(II) complexes. For this, we rely on a series of ligands with a different steric hindrance (Scheme 1) [16,17,18]. In this contribution, six Pd(II) complexes named as PtPzTn, PdPzTz, PdDMPzTn, PdDMPzTz, PdDPhPzTn and PdDPhPzTz, which formula is [PtCl_2_(L)], were successfully synthesized, structurally characterized and their in vitro cytotoxic activity measured. The aim of the study was to check the potential anticarcinogenic ability of these complexes in Hela, HL-60 and U937 cell lines via MTS (3-[4,5-dimethylthiazole-2-yl]-2,5-diphenyltetrazolium bromide) method and verify if the presence of bulky ligands influences the biological activity. Moreover, for the complex with better results, the accumulation of Pd(II) ion via ICP-MS and the induction of apoptosis by flow cytometry were checked.

## 2. Materials and Methods

### 2.1. Reagents and Instrumentation

The human histiocytic lymphoma (U-937), human promyelocytic leukemia (HL- 60), and human epithelial cervix adenocarcinoma (HeLa) cell lines were purchased from the European Collection of Authenticated Cell Cultures (ECACC; Dorset, UK). All chemical reagents were acquired from commercial sources. The synthesis of the ligands 2-(1-pyrazolyl)-2-thiazoline (PzTn), 2-(3,5-dimethyl-1-pyrazolyl)-2-thiazoline (DMPzTn), 2-(3,5-diphenyl-1-pyrazolyl)-2-thiazoline (DPhPzTn), 2-(1-pyrazolyl)-1,3-thiazine (PzTz), 2-(3,5-dimethyl-1-pyrazolyl)-1,3-thiazine (DMPzTz) and 2-(3,5-diphenyl-1-pyrazolyl)-1,3-thiazine (DPhPzTz) was carried out as reported elsewhere [16,17,18].

The elemental analysis was performed with a microanalyzer (Leco CHNS-932). IR spectra were recorded on a Perkin-Elmer 100 FTIR spectrophotometer from KBr pellets in the 4000–400 cm^−1^ range. To obtain ^1^H NMR spectra, it was used a Bruker 48 instrument at 300 MHz for PdPzTn and PdPzTz, an AV300 instrument at 300 MHz for PdDMPzTn, and a Bruker Avance III 500 instrument at 500 MHz for PdDPhPzTn and PdDPhPzTz, in DMF-d_7_ or DMSO-d_6_ according to the complex solubility. ^1^H NMR data could not be obtained for PdDMPzTz due to its low solubility. ^1^H NMR signals were referenced to residual proton resonances in deuterated solvents.

### 2.2. [PdCl_2_L] Synthesis

A mixture of the corresponding ligand and Na_2_[PdCl_4_]·H_2_O, both previously solved in ethanol (final volume 40 mL), was stirred at room temperature for 24 h and then filtered to provide a powder. The crude product was recrystallized with DMF for PdPzTn and PdPzTz, with DMSO for PdDMPzTn and PdDMPzTz and with acetonitrile for PdDPhPzTn and PdDPhPzTz. Crystals suitable for X-ray diffraction were gather up by filtration and washed with distilled water and cold diethyl ether.

#### 2.2.1. Synthesis of [PdCl_2_(PzTn)] (PdPzTn)

PzTn (100.0 mg, 0.7 mmol) and Na_2_[PdCl_4_]·H_2_O (192.0 mg, 0.7 mmol) were used to synthesize PdPzTn. Yield 189.8 mg (88.0%). Elemental analysis (%): cal. for C_6_H_7_Cl_2_PdN_3_S: C, 21.80; H, 2.14; N, 12.72; S, 9.70%. Found: C, 21.73; H, 2.08; N, 12.53; S, 9.39%. Selected IR data (KBr, cm^−1^): thiazoline ring vibration 1608 [υ(C=N)]; pyrazole ring vibrations 1532, 1413, 1371, 1001. ^1^H NMR (300 MHz, DMF-d_7_) δ 8.92 (d, 1H, J = 3.3 Hz), 8.16 (d, 1H, J = 2.1 Hz), 6.99 (dd, 1H, J = 2.1, 2.1 Hz), 4.30 (t, 2H, J = 7.7 Hz), 4.13 (t, 2H, J = 8.1 Hz), ppm.

#### 2.2.2. Synthesis of [PdCl_2_(PzTz)] (PdPzTz)

PzTz (100.0 mg, 0.6 mmol) and Na_2_[PdCl_4_]·H_2_O (176.0 mg, 0.6 mmol) were used to synthesize PdPzTz. Yield 175.5 mg (85.2%). Elemental analysis (%): cal. for C_7_H_9_Cl_2_PdN_3_S: C, 24.39; H, 2.64; N, 12.20; S, 9.30%. Found: C, 24.52; H, 2.65; N, 12.10; S, 9.28%. Selected IR data (KBr, cm^−1^): thiazine ring vibration 1596 [υ(C=N)]; pyrazole ring vibrations 1524, 1413, 1358, 1003. ^1^H NMR (300 MHz, DMF-d_7_) δ 8.81 (d, 1H, J = 3.0 Hz), 8.14 (d, 1H, J = 2.1 Hz), 6.92 (dd, 1H, J = 2.1, 2.1 Hz), 3.98 (t, 2H, J = 3.7 Hz), 3.58 (t, 2H, J = 5.7 Hz), 2.22 (m, 2H) ppm.

#### 2.2.3. Synthesis of [PdCl_2_(DMPzTn)] (PdDMPzTn)

DMPzTn (100.0 mg, 0.6 mmol) and Na_2_[PdCl_4_]·H_2_O (162.4 mg, 0.6 mmol) were used to synthesize PdDMPzTn. Yield 199.4 mg (95.3%). Elemental analysis (%): cal. for C_8_H_11_Cl_2_PdN_3_S: C, 26.79; H, 3.10; N, 11.73; S, 8.94%. Found: C, 26.83; H, 3.08; N, 11.34; S, 9.24%. Selected IR data (KBr, cm^−1^): thiazoline ring vibration 1601 [υ(C=N)]; pyrazole ring vibrations 1571, 1413, 1397, 1378, 973. ^1^H NMR (300 MHz, DMSO-d_6_) δ 6.60 (s, 1H), 4.22 (t, 2H, J = 8.4 Hz), 3.98 (t, 2H, J = 8.4 Hz), 2.65 (s, 3H), 2.63 (s, 3H), ppm.

#### 2.2.4. Synthesis of [PdCl_2_(DMPzTz)] (PdDMPzTz)

DMPzTz (100.0 mg, 0.5 mmol) and Na_2_[PdCl_4_]·H_2_O (150.7 mg, 0.5 mmol) were utilized to synthesize PdDMPzTz. Yield 158.2 mg (77.9%). Elemental analysis. (%): cal. for C_9_H_13_Cl_2_PdN_3_S: C, 29.03; H, 3.52; N, 11.29; S, 8.60%. Found: C, 28.85; H, 3.57; N, 11.17; S, 8.71%. Selected IR data (KBr, cm^−1^): thiazine ring vibration 1592 [υ(C=N)]; pyrazole ring vibrations 1564, 1404, 1382, 1341, 991.

#### 2.2.5. Synthesis of [PdCl_2_(DPhzTn)] (PdDPhPzTn)

DPhPzTn (100.0 mg, 0.3 mmol) and Na_2_[PdCl_4_]·H_2_O (96.4 mg, 0.3 mmol) were used to synthesize PdDPhPzTn. Yield: 76.2 mg (48.2%). Elemental analysis (%): cal. for C_18_H_15_Cl_2_PdN_3_S: C, 44.78; H, 3.14; N, 8.71; S, 6.64%. Found: C, 44.59; H, 3.02; N, 8.76; S, 6.67%. Selected IR data (KBr, cm^−1^): thiazoline ring vibration 1587 [υ(C=N)]; pyrazole ring vibrations 1556, 1411, 1314, 996. ^1^H NMR (500 MHz, DMF-d_7_) δ 7.84 (m, 4H), 7.66 (m, 3H), 7.48 (m, 3H), 7.12 (s, 1H), 4.13 (t, 2H, J = 8.5 Hz), 3.78 (t, 2H, J = 8.5 Hz) ppm.

#### 2.2.6. Synthesis of [PdCl_2_(DPhPzTz)] (PdDPhPzTz)

DPhPzTz (100.0 mg, 0.3 mmol) and Na_2_[PdCl_4_]·H_2_O (92.2 mg, 0.3 mmol) were used to synthesize PdDPhPzTz. Yield: 123.9 mg (79.7%). Elemental analysis (%): cal. for C_19_H_17_Cl_2_PdN_3_S: C, 45.93; H, 3.46; N, 8.47; S, 6.45%. Found: C, 45.90; H, 3.41; N, 8.92; S, 6.24%. Selected IR data (KBr, cm^−1^): thiazine ring vibration 1606 [υ(C=N)]; pyrazole ring vibrations 1556, 1404, 1313, 1000. ^1^H NMR (500 MHz, DMF-d_7_) δ 7.86 (m, 4H), 7.63 (m, 3H), 7.47 (m, 3H), 7.07 (s, 1H), 4.02 (t, 2H, J = 5.5 Hz), 3.28 (t, 2H, J = 6.0 Hz), 2.09 (m, 2H) ppm.

### 2.3. X-Ray Crystallography

Crystallographic data and experimental information of the six complexes are summarized in Table 1 and Table 2.

X-ray data were collected in a Bruker D8 VENTURE PHOTON III-14 diffractometer using graphite monochromated Mo Kα radiation (λ = 0.71073 Å) and the ω scan technique at 100 K. The collected frames were processed with the SAINT [19] and corrected for absorption using SADABS program [20]. The structures were solved by direct method using the SHELXS-14 [21] program and refined by full-matrix least-squares on F2 with SHELXL-18 [22], included in WINGX [23] package. All non-hydrogen atoms were refined with anisotropic displacement parameters. Hydrogen atoms were placed at calculated positions and refined using a riding model. Crystallographic data for palladium(II) complexes have been deposited at the Cambridge Crystallographic Data Centre, CCDC Nos. 2224537, 2224538, 2224539, 2224540, 2224541 and 2224542. These data can be obtained free of charge from the Cambridge Crystallographic Data Centre via www.ccdc.cam.ac.uk/data_request/cif (accessed on 3 January 2023).

### 2.4. Treatment Conditions

Human cancer cell lines (HeLa, HL-60 and U-937) were challenged with the free ligands DMPzTn and DMPzTz (PzTn, PzTz, DPhPzTn and DPhPzTz were tested in previous studies [15]) and the Pd(II) complexes (PdPzTn, PdDMPzTn, PdDPhPzTn, PdPzTz, PdDMPzTz or PdDPhPzTz) at concentrations ranging from 1–100 µM for 24 h. Further experiments at 48 and 72 h were performed at the same concentrations of the complexes in HeLa cells. To prepare PdPzTn, PdPzTz, PdDPhPzTn and PdDPhPzTz solutions, dimethylformamide (DMF) was used, while dimethyl sulfoxide (DMSO) was used for PdDMPzTn and PdDMPzTz, obtaining in these cases suspensions due to the low solubility of both complexes. To take into account the solvent effect, a control with DMF or DMSO was used (vehicle), being the final concentrations of these solvents lower than 0.5% (*v*/*v*).

### 2.5. Cytotoxicity

CellTiter 96 AQueous One Solution Cell Proliferation Assay (Promega, Madrid, Spain) was used to estimate the cytotoxicity of the complexes synthesized on the tumor cell lines selected. This assay is based on the reduction of an MTS tetrazolium compound. To perform the experiment, HeLa, U-937, and HL-60 cells were seeded in 96-well plates (8 × 10^3^, 1.5 × 10^4^, 2.5 × 10^4^ cells/well, respectively), and challenged with different concentrations of the compounds for 24, 48, or 72 h. After that time, 10 µL of the CellTiter 96 AQueous One Solution Reagent were added directly to culture wells and then incubated at 37 °C at different times according to the cell type (15 min, 1 h, and 2 h for HeLa, U-937, and HL-60, respectively). In the end, a microplate reader (Infinite M200; Tecan, Grödig, Austria) was used to record the absorbance at $\lambda_{test}=490\;nm$ and $\lambda_{reference}=650\;nm$. All experiments were performed in triplicate. The percentage of cell viability was normalized to control samples.

### 2.6. Apoptosis

A commercial kit, which is based on annexin V-FITC and propidium iodide double-staining (ThermoFisher Scientific, Barcelona, Spain), was employed to measure the induction of apoptosis, as reported elsewhere [24].

### 2.7. Palladium Uptake

To verify whether the Pd(II) accumulated inside HeLa cells and to which extent, a density of 2 × 10^6^ cells were seeded in 100 mm Petri dishes and treated with 67.55 µM of PdPzTn, 77.75 µM of PdPzTz, 62.74 µM of PdDPhPzTn or 57.83 µM of PdDPhPzTz for 4 h. After trypsinization and washing with PBS, cells were centrifuged at 500× *g* for 5 min and then lysed with RIPA buffer [15]. Finally, after digestion with 65% HNO_3_, cells were analyzed with an ICP-MS 7900 (Agilent Technologies, Madrid, Spain).

### 2.8. Analysis of Statistical Data

Statistical differences between measured variables were calculated by one-way ANOVA. Subsequent between-group comparisons were examined using the Tukey or Dunnett test, as indicated. Nonlinear regression was performed to determine IC_50_ values. *p* < 0.05 was considered to indicate a statistically significant difference. GraphPad Prism 7.04 statistics software (GraphPad Software, San Diego, CA, USA) was used.

## 3. Results and Discussion

### 3.1. Chemistry

The Pd(II) complexes synthesis was carried out as described in Scheme 1. To sum up the process, they were prepared by making the corresponding ligands react with Na_2_[PdCl_4_]·H_2_O in an ethanol solution under agitation for 24 h. Complexes’ characterization was made using single-crystal X-ray diffraction analysis, elemental analysis, IR spectroscopy, and ^1^H NMR spectroscopy.

### 3.2. X-Ray Crystal Structures

**Figure 1:**
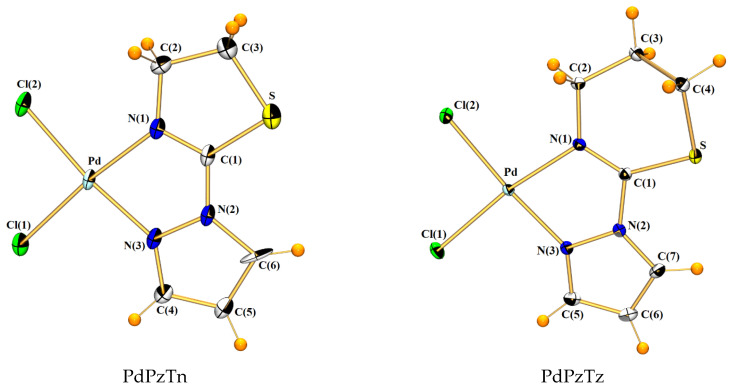

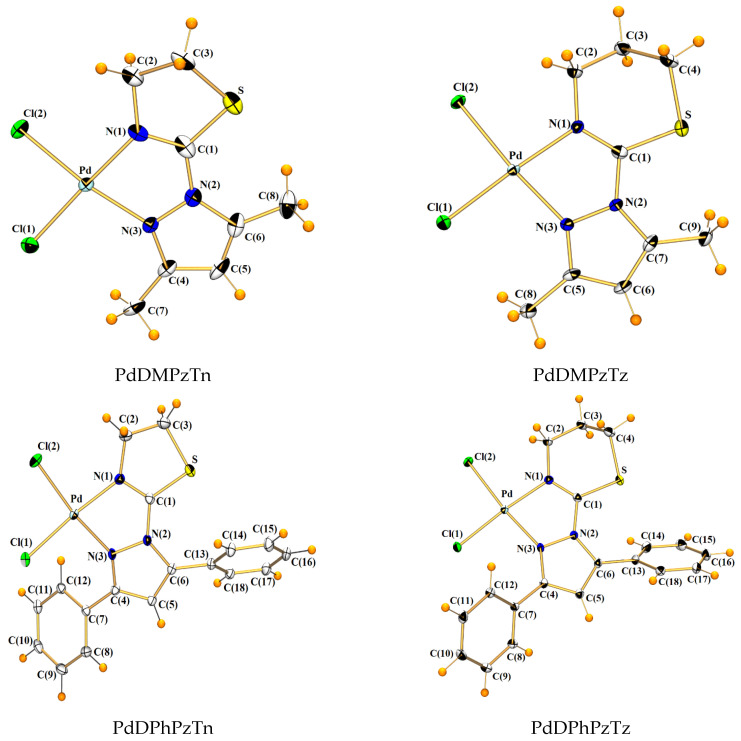
Crystal structures of PdPzTn, PdPzTz, PdDMPzTn, PdDMPzTz, PdDPhPzTn, and PdDPhPzTz.

Crystal structures of Pd(II) complexes consist of monomeric units [PdCl_2_L] (L = PzTn, PzTz, DMPzTn, DMPzTz, and DPhPzTn) or [PdCl_2_L]·C_2_H_3_N (L = DPhPzTz,). Crystal structures of all complexes can be observed in Figure 1. All Pd(II) complexes show a square planar geometry around the metal center, with different grades of distortion. Thus, PdPzTn does not present distortion, whereas the remaining compounds show a slight deviation from the square planar coordination sphere, being the dihedral angles between Cl(1)-Pd-Cl(2) and N(1)-Pd-N(3) lower than 8° (Table S1). The Pd(II) is surrounded by all donating, two chlorines in cis disposition and two nitrogen atoms of the organic ligand molecule. In Table 3 and Table 4, the selected bond distances and angles can be found.

Table 3 shows that the Pd-Cl bond lengths are similar in all complexes and longer than the Pd-N bond lengths. After checking the data obtained from the Cambridge Structural Database (CSD, Version v5.42, September 2022) [25] for square planar cis-complexes with a Cl_2_N_2_ coordination environment around Pd(II), it can be affirmed that all the Pd-Cl distances are similar to the mean values found [2.294(19) Å for 944 Pd(II) complexes], like what happens for Pd-N_pyrazole_ bond distances with respect to the mean value calculated [2.031(19) Å for 109 Pd(II) complexes] in CSD [25]. As for the Pd-N_thiazoline_, bond distances are slightly shorter than the average values calculated for this type of bond in CSD [25]: 2.037(59) Å for 14 Pd(II) complexes, unlike Pd-N_thiazine_ lengths, which are barely larger than the only complex found in CSD: 2.0160 Å [14].

With respect to the arrangement of the organic ligands in the Pd(II) complexes, it can be observed that the thiazoline and thiazine rings are rotated around the C(1)-N(2) bond so that the N(1) and N(3) atoms to simultaneously coordinate to the Pd(II) ions. This can be confirmed by the data corresponding to the torsion angles of the complexes and the free ligands shown in Table S2.

Moreover, the crystal structures are stabilized by the parallel displaced aromatic interactions between phenyl rings in both PdDPhPzTn and PdDPhPzTz complexes and between pyrazole rings in PdPzTz. On the other hand, π–π stacking interactions are produced in both PdDMPzTn and PdDMPzTz complexes. A representation of these interactions is shown in Figure S1, and the values are specified in Table S7.

### 3.3. Spectroscopic Studies

A comparison of the ^1^H NMR spectral data for PdPzTn, PdPzTz, PdDMPzTn, PdDPhPzTn, and PdDPhPzTz and their respective ligands are presented in Tables S5 and S6. It is noted that ^1^H NMR signals of thiazine and thiazoline of all complexes are in general shifted to higher frequencies with respect to their free ligands. This also occurs for pyrazole ring signals for PdPzTn, PdPzTz, and PdDMPzTn, while the signals of complexes with phenyl substituents are slightly shifted to lower frequencies, probably due to the presence of aromatic groups which help with the redistribution of electronic density loss [26,27]. Therefore, it can be concluded that the ligands are coordinated to palladium in DMF solution for PdPzTn, PdPzTz, PdDPhPzTn, and PdDPhPzTz and in DMSO solution for PdDMPzTn. To confirm the stability of the complexes in the solution, they were kept in DMF or DMSO for 21 days. After that time, the ^1^H NMR was measured again (data not included), remaining intact, thus proving that the complexes have high stability in the solution. In Figures S8–S17, the ^1^H NMR spectra can be found.

On the other hand, the IR spectra of complexes PdPzTn, PdPzTz, PdDMPzTn, PdDMPzTz, PdDPhPzTn, and PdDPhPzTz (Figures S2–S7 and Tables S3 and S4) showed that the bands attributable to W_1_[υ(C=N)] vibration in thiazoline and Ψ_1_[υ(C=N)] vibration in thiazine suffers a shift to lower wavenumber in the complexes with respect to their respective ligands. It has been described that the reason for this displacement is related to the presence of the C-N bond as part of a chelate ring where the back-coordination from the metal to the ligand compensates for the loss of electron density on the nitrogen atom. In addition, the bands assigned to pyrazole ring vibrations are shifted to a higher wavenumber for PdPzTn and PdPzTz, not showing so well define patron for the other four complexes, which can be explained by the presence of the substituents. So, the palladium coordination to both heterocycles through the nitrogen atoms was confirmed for these frequency shifts in the infrared spectra [17,18].

### 3.4. Biological Studies

**Figure 2:**
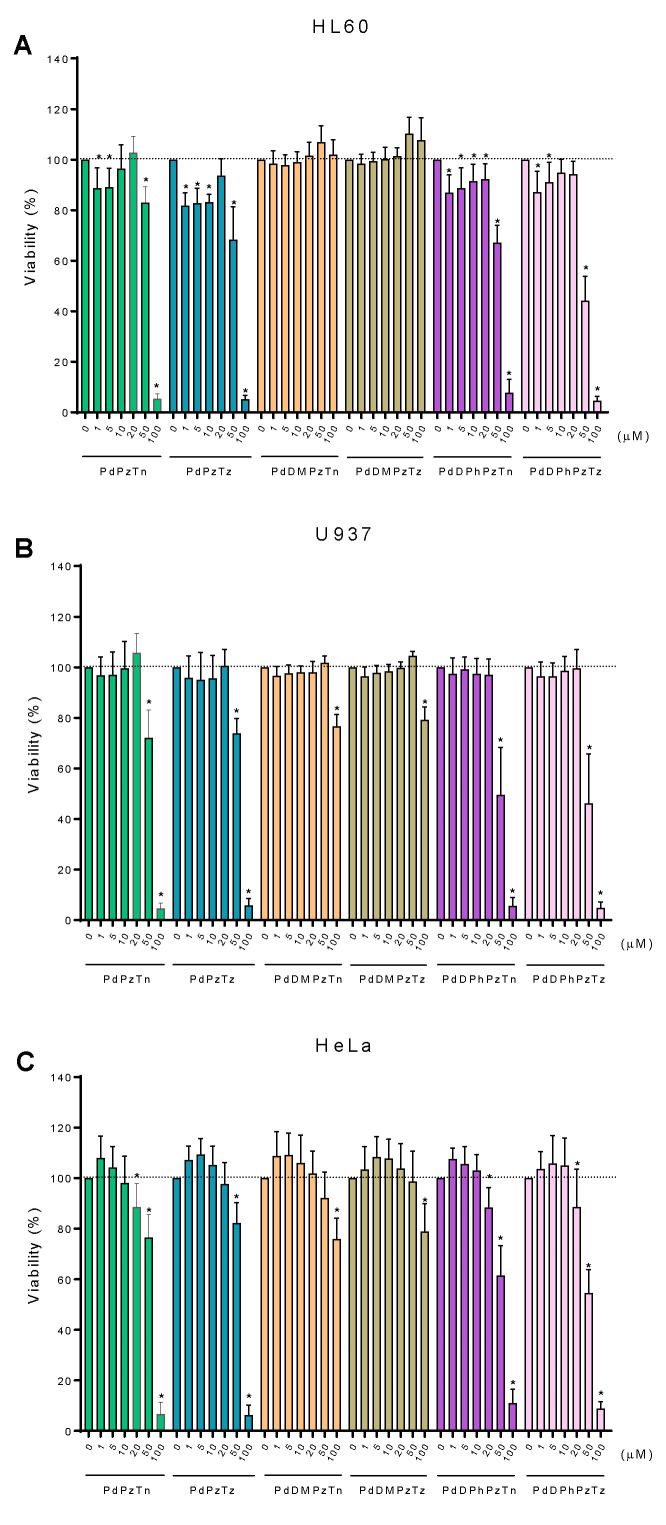
Cytotoxic effect of Pd(II) complexes. Promyelocytic leukemia HL-60 (**A**), histiocytic lymphoma U-937 (**B**), and epithelial cervix carcinoma HeLa (**C**) cell lines were challenged with 1, 5, 10, 20, 50, and 100 μM of the six Pd(II) complexes, namely PdPzTn, PdPzTn, PdDMPzTn, PdDMPzTz, PdDPhPzTn, and PdDPhPzTz, or the vehicle (DMF or DMSO) for 24 h. Values are presented as means ± S.D. (*n* = 6) and depicted as percentage of untreated samples. * *p* < 0.05 vs. control (0 μM).

In order to verify the cytotoxicity of the compounds, Pd(II) complexes were tested at concentrations from 1 to 100 µM on the three tumor cell lines selected during 24 h (Figure 2). Data obtained revealed that PdPzTn, PdPzTz, PdDPhPzTn, and PdDPhPzTz had a moderate cytotoxic effect on all three cell lines studied. On the contrary, PdDMPzTn and PdDMPzTz did not show cytotoxic effects until high concentrations (IC_50_ values > 100 µM) (Figure 2A–C and Table 5). Moreover, complexes with the presence of ligands with phenyl groups (PdDPhPzTn, PdDPhPzTz) showed lower IC_50_ values than the analogs without extra substituents, improving the cytotoxicity of the compounds. The IC_50_ values found for these two complexes were between 46.39 ± 3.99 μM and 62.74 ± 6.45 μM in the three lines, with no existing strong differences between the complexes or the cell lines (Figure 2A–C and Table 5). As reported in previous publications, other palladium(II) complexes have been studied on tumor cells, but in general with longer incubation times (48–72 h) [28,29,30,31,32]. To properly compare our findings with the literature, the cytotoxicity assay was also performed at 48 and 72 h in HeLa cells. The results obtained showed a reduction of the IC_50_ values in complexes PdPzTn, PdPzTz, PdDPhPzTn, and PdDPhPzTz, unlike PdDMPzTn and PdDMPzTn, which did not improve their effect along time (Table S8). The decrease in the IC_50_ values was around 10 μM for PdPzTn and PdPzTz after 48 h of treatment, and between 25–30 μM at 72 h. These results are even better for both complexes with phenyl groups whose IC_50_ values decreased more than 25 μM at 48 h and between 30–40 μM at 72 h. Therefore, the better results obtained were those of PdDPhPzTn and PdDPhPzTz after 72 h of treatment, with IC_50_ values of 23.28 ± 2.44 μM and 27.98 ± 3.33 μM, respectively. It could be concluded that, in general, longer incubation times improved the cytotoxic effect of most of our complexes.

With respect to other palladium(II) complexes reported, some of them have better biological results than ours at the same incubation times [28,30,32]. In contrast, our results improved some of the IC_50_ values achieved with other palladium(II) compounds, thereby decreasing the concentration of some of them at the same incubation times, and even having lower IC_50_ at shorter incubation times (24 h) [29,31,32]. Moreover, compared to closer structures to the ones presented in this work, we were not able to improve the biological activity of a complex previously synthesized by our lab (PdTdTn) on the U937 cell line [13]. In contrast, for other palladium(II) complexes already reported by our lab (PdPyTz), it was possible to improve the IC_50_ values in all three cell lines used in the present work [14]. Particularly, the complexes with phenyl groups decrease the IC_50_ in the HL-60 cell line at 50 µM.

On the other hand, it was checked that none of the free ligands generated relevant cytotoxicity on any of the tumor cell lines studied, except for DPhPzTn and DPhPzTz in HeLa cells at high doses (Figure S18 and [15]). As it was not seen any special sensitivity of the cell lines neither for the complexes nor the ligands, solid tumor HeLa cells were selected as a model to perform subsequent experiments.

ICP-MS was used to verify Pd(II) accumulation into HeLa cells. To do that, the tumor cells were incubated the IC_50_ of PdPzTn, PdPzTz, PtDPhPzTn, and PdDPhPzTz during 4 h. Complexes with ligands substituted with methyl groups were not tested due to the lack of relevant cytotoxic activity (Figure 2A–C and Table 5). As it can be seen in Table 6, an accumulation of the metal ion was produced after incubation with the four compounds. However, PdDPhPzTn and PdDPhPzTz showed a more efficient accumulation into cells than their analogs, although any significant difference was found. Even so, the higher accumulation and lower IC_50_ values of the two complexes with phenyl rings might indicate that the presence of aromatic groups in the ligands improved their cellular uptake. This could be related with a presumable higher lipophilicity for PdDPhPzTn and PdDPhPzTz, since they showed the same trend that we found for platinum(II) complexes with the same ligands in previous studies [15].

**Figure 3:**
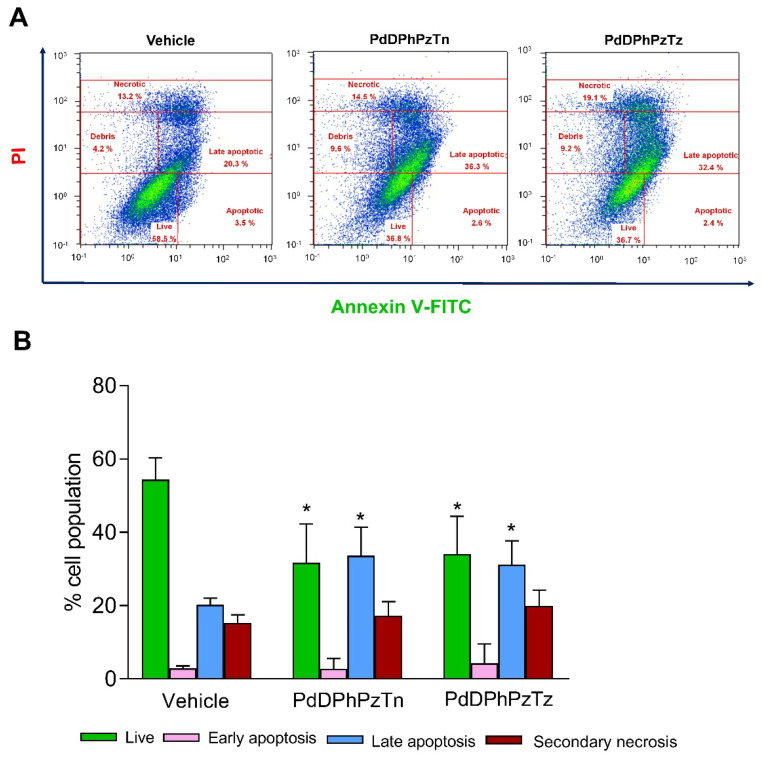
Apoptotic cell death caused by palladium complexes. IC_50_ of PdDPhPzTn (57.83 µM), PdDPhPzTz (62.74 µM) or the vehicle (1.5% DMF) were tested on HeLa cells for 24 h. (**A**) Illustrative cytograms of annexin V-FITC-stained cells in the presence of propidium iodide (PI). (**B**) Histogram bars show percentages of live, early apoptotic, late apoptotic, and secondary necrotic cells detected by flow cytometry. Values are presented as means ± SD of four independent experiments. * *p* < 0.05 compared to control values (Dunnett’s test).

Finally, to check whether the reduction in cell viability showed by PdDPhPzTn and PdDPhPzTz was mediated by the apoptosis, flow cytometry experiments using annexin V-FITC and PI were carried out. The treatment of HeLa cells with IC_50_ of the complexes with phenyl rings during 24 h produced significant changes in the percentage of cell populations, particularly in the case of live (decrease) and late apoptotic (increase) cells for both complexes, in comparison to the vehicle-treated (1.5% DMF; Figure 3) and untreated (control) cells (Figure S19). These results suggested that the major type of cell death prompted by PdDPhPzTn and PdDPhPzTz was apoptosis.

## 4. Conclusions

In this study, we have synthesized and characterized six new Pd(II) complexes with ligands containing pyrazole and thiazine/thiazoline heterocycles. The cytotoxic effect produced by these complexes was checked in three different tumor cell lines (HeLa, HL-60, and U937). Our results showed no modification of cell viability for complexes with methyl substituents on any of the three lines and not even at longer incubation times. On the other hand, moderate cytotoxicity was found for complexes with no extra substituents or with phenyl rings, in both cases improving the biological effects of their respective ligands. Interestingly, longer incubation times strongly improved the cytotoxic effects of these complexes, thereby reducing their IC_50_ values. Particularly, lower IC_50_ values, proapoptotic effects, and higher cellular uptake of Pd(II) were observed for PdDPhPzTz and PdDPhPzTn. Thus, the presence of aromatic rings in the ligand may influence the accumulation and biological activity of these complexes, improving their effectivity in solid and non-solid tumors.

## Figures and Tables

**Table 1:** Crystal information, data collection, and refinement details for PdPzTn. PdPzTz, PdDMPzTn, and PdDMPzTz.

	PdPzTn	PdPzTz	PdDMPzTz	PdDMPzTz
Crystal shape	Needle	Plate	Needle	Needle
Color	Yellow	Orange	Yellow	Yellow
Size (mm)	0.16 × 0.05 × 0.03	0.19 × 0.18 × 0.06	0.25 × 0.02 × 0.02	0.24 × 0.03 × 0.02
Chemical formula	C_6_H_7_Cl_2_N_3_PdS	C_7_H_9_Cl_2_N_3_PdS	C_8_H_11_Cl_2_N_3_PdS	C_9_H_13_Cl_2_N_3_PdS
Formula weight	330.51	344.53	358.56	372.58
Crystal system	Monoclinic	Monoclinic	Monoclinic	Orthorhombic
Space group	C 2/m	P 2_1_/n	P 2_1_/c	P b c a
Unit cell dimensions				
*a* (Å)	16.317 (3)	8.9806 (5)	17.9491 (18)	7.4256 (4)
*b* (Å)	6.7035 (10)	7.3615 (4)	7.2963 (7)	17.6074 (12)
*c* (Å)	8.7925 (14)	16.2772 (10)	17.5523 (15)	18.3241 (13)
*α* (°)	90	90	90	90
*β* (°)	95.489 (9)	101.738 (2)	91.305 (3)	90
*γ* (°)	90	90	90	90
Cell volume (Å^3^)	957.3 (3)	1053.59 (10)	2298 (14)	2395.8 (3)
Z	4	4	8	8
D_calc_ (g cm^−3^)	2.293	2.172	2.073	2.066
*µ* (mm^−1^)	2.666	2.427	2.23	2.143
F (000)	640	672	1408	1472
θ range	2.327–30.494	2.407–30.529	2.27–28.439	2.313–26.369
Index ranges	−23 ≤ h ≤ 23, −9 ≤ k ≤ 9, −12 ≤ l ≤ 12	−12 ≤ h ≤ 12, −10 ≤ k ≤ 10, −23 ≤ l ≤ 23	−24 ≤ h ≤ 23, −0 ≤ k ≤ 9, −0 ≤ l ≤ 23	−9 ≤ h ≤ 9, −22 ≤ k ≤ 22, −22 ≤ l ≤ 22
Temperature	100	101	100	100
Independent reflections	1579	3228	10,104	2448
Observed reflections	1506	3106	7828	2112
No. of refined parameters	79	127	272	147
R [F > 4.0 σ (F)]	0.0243	0.0161	0.059	0.0335
wR [F > 4.0 σ (F)]	0.0643	0.0503	0.1311	0.0786
GOF	0.875	1.057	1.06	1.087
ρ*_max_*, ρ*_min_* (e Å^−3^)	1.777, −1.155	0.413, −0.772	1.325, −1.441	0.671, −0.781

**Table 2:** Crystal information, data collection, and refinement details for PdDPhPzTn and PdDPhPzTz.

	PdDPhPzTn	PdDPhPzTz
Crystal shape	Prism	Prism
Color	Orange	Orange
Size (mm)	0.12 × 0.11 × 0.08	0.13 × 0.12 × 0.11
Chemical formula	C_18_H_15_Cl_2_N_3_PdS	C_19_H_17_Cl_2_N_3_PdS·C_2_H_3_N
Formula weight	482.69	537.77
Crystal system	Triclinic	Triclinic
Space group	P$\bar{1}$	P$\bar{1}$
Unit cell dimensions		
*a* (Å)	9.5024 (6)	9.498 (3)
*b* (Å)	9.7838 (6)	11.092 (4)
*c* (Å)	11.4434 (7)	11.502 (4)
*α* (°)	76.448 (3)	66.569 (13)
*β* (°)	71.639 (3)	77.150 (13)
*γ* (°)	62.954 (3)	74.427 (13)
Cell volume (Å^3^)	894.07 (10)	1061.9 (6)
Z	2	2
D_calc_ (g cm^−3^)	1.793	1.682
*µ* (mm^−1^)	1.459	1.239
F (000)	480	540
θ range	2.479–36.316	2.245–36.316
Index ranges	−15 ≤ h ≤ 15, −16 ≤ k ≤ 16, −19 ≤ l ≤ 19	−15 ≤ h ≤ 15, 18 ≤ k ≤ 18, −19 ≤ l ≤ 19
Temperature	100	100
Independent reflections	8668	10,285
Observed reflections	6943	8316
No. of refined parameters	226	163
R [F > 4.0 σ (F)]	0.0349	0.034
wR [F > 4.0 σ (F)]	0.0742	0.0797
GOF	1.028	0.987
ρ*_max_*, ρ*_min_* (e Å^−3^)	0.673, −1.689	0.735, −1.415

**Table 3:** Selected bond distances (Å).

	Pd-Cl(1)	Pd-Cl(2)	Pd-N(1)	Pd-N(3)
PdPzTn	2.278(1)	2.299(1)	2.008(3)	2.009(3)
PdPzTz	2.292(0)	2.294(0)	2.030(1)	1.996(1)
PdDMPzTn	2.280(2)	2.304(2)	1.999(7)	2.036(6)
PdDMPzTz	2.289(1)	2.285(1)	2.025(3)	2.007(3)
PdDPhPzTn	2.283(1)	2.286(0)	2.005(2)	2.036(1)
PdDPhPzTz	2.291(1)	2.283(1)	2.031(2)	2.026(2)

**Table 4:** Selected angles (°).

	Cl(2)-Pd-Cl(1)	N(1)-Pd-Cl(1)	N(1)-Pd-Cl(2)	N(3)-Pd-Cl(1)	N(3)-Pd-Cl(2)
PdPzTn	92.76(4)	93.43(9)	173.82(8)	172.93(9)	94.31(9)
PdPzTz	92.41(1)	95.14(4)	171.42(4)	174.84(3)	92.55(3)
PdDMPzTn	88.73(8)	93.3(2)	177.30(19)	172.3(2)	98.8(2)
PdDMPzTz	88.21(4)	94.90(9)	175.03(9)	174.23(9)	97.26(9)
PdDPhPzTn	90.53(2)	93.45(4)	174.03(4)	171.67(4)	97.37(4)
PdDPhPzTz	89.08(3)	94.78(5)	174.06(4)	171.79(4)	97.19(5)

**Table 5:** Cytotoxic effect of PdPzTn, PdPzTz, PdDMPzTn, PdDMPzTz, PdDPhPzTn, and PdDPhPzTz towards selected tumor cell lines.

	HeLa	HL-60	U-937
PdPzTn	67.55 ± 7.27 ^a^	71.81 ± 9.09 ^a^	70.95 ± 8.73 ^a^
PdPzTz	77.75 ± 9.68 ^a^	54.50 ± 6.68 ^a^	70.21 ± 7.67 ^a^
PdDMPzTn	>150 ^b^	>150 ^b^	>150 ^b^
PdDMPzTz	>150 ^c^	>150 ^b^	>150 ^c^
PdDPhPzTn	62.74 ± 6.45 ^a^	58.83 ± 4.94 ^a^	53.43 ± 4.91 ^a^
PdDPhPzTz	57.83 ± 6.45 ^a^	46.39 ± 3.99 ^a^	50.35 ± 4.82 ^a^

For a given cell line, a different lowercase letter indicates statistically significant (*p* < 0.05) changes (Tukey’s test). Data are presented as IC_50_ ± SD (µM).

**Table 6:** Palladium accumulation in HeLa cells.

	Pd (Means ± SD; ng/mL)
PdPzTn	9.05 ± 5.66
PdPzTz	10.38 ± 6.61
PdDPhPzTn	14.57 ± 10.69
PdDPhPzTz	15.36 ± 7.98

*n* = 4.

## Data Availability

The data are contained within this article and the Supplementary File.

## Supplementary Materials

The following supporting information can be downloaded at: https://www.mdpi.com/article/10.3390/pharmaceutics15020696/s1, Table S1: Dihedral angles (°) between the planes Cl(1)-Pd-Cl(2) and N(1)-Pd-N(3) in PdPzTn, PdPzTz, PdDMPzTn, PdDMPzTz PdDPhPzTn and PdDPhPzTz complexes; Table S2: Torsion angles (°) for PdPzTn, PdPzTz, PdDMPzTn, PdDMPzTz PdDPhPzTn and PdDPhPzTz complexes and their respective ligands; Table S3: IR spectral assignments (cm^−1^) for PzTn, PdPzTn, DMPzTn, PdDMPzTn, DPhPzTn, and PdDPhPzTn; Table S4: IR spectral assignments (cm^−1^) for PzTz, PdPzTz, DMPzTz, PdDMPzTz, DPhPzTz, and PdDPhPzTz; Table S5: ^1^H NMR spectral data for PzTn, PdPzTn, DPhPzTn and PdDPhPzTn complexes in DMF-d_7_ solvent and for DMPzTn and PdDMPzTn in DMSO-d_6_; Table S6: ^1^H NMR spectral data for PzTz, PdPzTz, DPhPzTz and PdDPhPzTz complexes in DMF-d_7_; Table S7: Aromatic interactions in PdPzTz, PdDMPzTn, PdDMPzTz, PdDPhPzTn and PdDPhPzTz; Table S8: Cytotoxicity (IC_50_ ± SD, µM) of the different Pd(II) complexes in HeLa cells after 48 and 72 h; Figure S1: Aromatic interactions in PdPzTz, PdDMPzTn, PdDMPzTz, PdDPhPzTn and PdDPhPzTz; Figure S2: IR spectrum of [PdCl_2_(PzTn)] in 4000–400 cm^−1^ region; Figure S3: IR spectrum of [PdCl_2_(PzTz)] in 4000–400 cm^−1^ region; Figure S4: IR spectrum of [PdCl_2_(DMPzTn)] in 4000–400 cm^−1^ region; Figure S5: IR spectrum of [PdCl_2_(DMPzTz)] in 4000–400 cm^−1^ region; Figure S6: IR spectrum of [PdCl_2_(DPhPzTn)] in 4000–400 cm^−1^ region; Figure S7: IR spectrum of [PdCl_2_(DPhPzTz)] in 4000–400 cm^−1^ region; Figure S8: ^1^H NMR spectrum of PzTn in DMF-d_7_; Figure S9: ^1^H NMR spectrum of PdPzTn in DMF-d_7_; Figure S10: ^1^H NMR spectrum of PzTz in DMF-d_7_; Figure S11: ^1^H NMR spectrum of PdPzTz in DMF-d_7_; Figure S12: ^1^H NMR spectrum of DMPzTn in DMSO-d_6_; Figure S13: ^1^H NMR spectrum of PdDMPzTn in DMSO-d_6_; Figure S14: ^1^H NMR spectrum of DPhPzTn in DMF-d_7_; Figure S15: ^1^H NMR spectrum of PdDPhPzTn in DMF-d_7_; Figure S16: ^1^H NMR spectrum of DPhPzTz in DMF-d_7_; Figure S17: ^1^H NMR spectrum of PdDPhPzTz in DMF-d_7_; Figure S18: Dose-response curves of the pyrazole/thiazoline ligands on cell viability. HL-60 (A), U-937 (B), and HeLa (C) cells were treated for 24 h with increasing concentrations (0–100 μM, as indicated) of the ligands DMPzTn and DMPzTz, or the vehicle (DMSO, control). Data represent means ± S.D. of 5 independent experiments and are expressed as percentage of control values. * *p* < 0.05 compared to their corresponding control values; Figure S19: Representative cytogram of untreated HeLa cells stained with annexin V-FITC in the presence of propidium iodide (PI).

## References

[1] WHO Cancer Geneva: World Health Organization. https://www.who.int/news-room/fact-sheets/detail/cancer.

[2] Fanelli M., Formica M., Fusi V., Giorgi L., Micheloni M., Paoli P. (2016). New Trends in Platinum and Palladium Complexes as Antineoplastic Agents. Coord. Chem. Rev..

[3] Dilruba S., Kalayda G.V. (2016). Platinum-Based Drugs: Past, Present and Future. Cancer Chemother. Pharmacol..

[4] Gou Y., Huang G.J., Li J., Yang F., Liang H. (2021). Versatile Delivery Systems for Non-Platinum Metal-Based Anticancer Therapeutic Agents. Coord. Chem. Rev..

[5] De la Cueva-Alique I., de la Torre-Rubio E., Muñoz-Moreno L., Calvo-Jareño A., Pérez-Redondo A., Gude L., Cuenca T., Royo E. (2022). Stereoselective Synthesis of Oxime Containing Pd(Ii) Compounds: Highly Effective, Selective and Stereo-Regulated Cytotoxicity against Carcinogenic PC-3 Cells. Dalt. Trans..

[6] Medici S., Peana M., Nurchi V.M., Lachowicz J.I., Crisponi G., Zoroddu M.A. (2015). Noble Metals in Medicine: Latest Advances. Coord. Chem. Rev..

[7] Vojtek M., Marques M.P.M., Ferreira I.M.P.L.V.O., Mota-Filipe H., Diniz C. (2019). Anticancer Activity of Palladium-Based Complexes against Triple-Negative Breast Cancer. Drug Discov. Today.

[8] Azzouzi A.R., Lebdai S., Benzaghou F., Stief C. (2015). Vascular-Targeted Photodynamic Therapy with TOOKAD^®^ Soluble in Localized Prostate Cancer: Standardization of the Procedure. World J. Urol..

[9] Alam M.N., Huq F. (2016). Comprehensive Review on Tumour Active Palladium Compounds and Structure–Activity Relationships. Coord. Chem. Rev..

[10] Dehand J., Jordanov J., Beck J.P. (1975). Anti-Tumour Activity of Heavy Transition Metal Complexes against Hepatoma Cells. Chem. Biol. Interact..

[11] Akhmetova V.R., Akhmadiev N.S., Abdullin M.F., Dzhemileva L.U., D’yakonov V.A. (2020). Synthesis of New N,N’-Pd(Pt) Complexes Based on Sulfanyl Pyrazoles, and Investigation of Their in Vitro Anticancer Activity. RSC Adv..

[12] Espino J., Fernández-Delgado E., Estirado S., de la Cruz-Martinez F., Villa-Carballar S., Viñuelas-Zahínos E., Luna-Giles F., Pariente J.A. (2020). Synthesis and Structure of a New Thiazoline-Based Palladium(II) Complex That Promotes Cytotoxicity and Apoptosis of Human Promyelocytic Leukemia HL-60 Cells. Sci. Rep..

[13] Fernández-Delgado E., de la Cruz-Martínez F., Galán C., Franco L., Espino J., Viñuelas-Zahínos E., Luna-Giles F., Bejarano I. (2020). Pt(II) and Pd(II) Complexes with a Thiazoline Derivative Ligand: Synthesis, Structural Characterization, Antiproliferative Activity and Evaluation of pro-Apoptotic Ability in Tumor Cell Lines HT-29 and U-937. J. Inorg. Biochem..

[14] Gutiérrez-Tarriño S., Espino J., Luna-Giles F., Rodríguez A.B., Pariente J.A., Viñuelas-Zahínos E., Crisponi G., Dominguez-Martin A. (2021). Synthesis, Characterization and Antiproliferative Evaluation of Pt(II) and Pd(II) Complexes with a Thiazine-Pyridine Derivative Ligand. Pharmaceuticals.

[15] Fernández-Delgado E., Estirado S., Espino J., Viñuelas-Zahínos E., Luna-Giles F., Rodríguez Moratinos A.B., Pariente J.A. (2022). Influence of Ligand Lipophilicity in Pt(II) Complexes on Their Antiproliferative and Apoptotic Activities in Tumour Cell Lines. J. Inorg. Biochem..

[16] Bernalte-García A., Lozano-Vila A.M., Luna-Giles F., Pedrero-Marín R. (2006). Structural Characterization of a Thiazoline-Pyrazole Ligand and Its Complexes with Cobalt(II) and Copper(II). Polyhedron.

[17] Torres-García P., Viñuelas-Zahínos E., Luna-Giles F., Espino J., Barros-García F.J. (2011). Zinc(II) Complexes with Novel 1,3-Thiazine/Pyrazole Derivative Ligands: Synthesis, Structural Characterization and Effect of Coordination on the Phagocytic Activity of Human Neutrophils. Polyhedron.

[18] Torres-García P., Pedrero-Marín R., Luna-Giles F., Huertas-Sánchez A.V.V., Viñuelas-Zahínos E. (2012). Influence of Steric Strain of S,N-Heterocycles Derivative Ligands on the Coordination Geometry in Cadmium(II) Nitrato Complexes. Polyhedron.

[19] Bruker (2015). APEX3 and SAINT.

[20] Bruker (2012). SADABS.

[21] Sheldrick M. (2014). SHELXS-14, Program for Crystal Structures Solution.

[22] Sheldrick G.M. (2015). Crystal Structure Refinement with SHELXL. Acta Crystallogr. Sect. C.

[23] Farrugia L.J. (2012). WinGX and ORTEP for Windows: An Update. J. Appl. Crystallogr..

[24] Espino J., Rodríguez A.B., Pariente J.A. (2013). The Inhibition of TNF-α-Induced Leucocyte Apoptosis by Melatonin Involves Membrane Receptor MT1/MT2 Interaction. J. Pineal Res..

[25] Bruno I.J., Cole J.C., Edgington P.R., Kessler M., Macrae C.F., McCabe P., Pearson J., Taylor R. (2002). New Software for Searching the Cambridge Structural Database and Visualizing Crystal Structures. Acta Crystallogr. Sect. B Struct. Sci..

[26] Ocansey E., Darkwa J., Makhubela B.C.E. (2019). Bis(Pyrazolyl)Palladium(II) Complexes as Catalysts for Mizoroki–Heck Cross-Coupling Reactions. Polyhedron.

[27] Zulu S., Alam M., Ojwach S.O., Akerman M.P., Stephen Ojwach C.O., Address P. (2019). Structural and Theoretical Studies of the Methoxycarbonylation of Higher Olefins Catalysed by (Pyrazolyl-Ethyl)Pyridine Palladium (II) Complexes. Appl. Organometal. Chem..

[28] Gligorijević N., Todorović T., Radulović S., Sladić D., Filipović N., Godevac D., Jeremić D., Andelković K. (2009). Synthesis and Characterization of New Pt(II) and Pd(II) Complexes with 2-Quinolinecarboxaldehyde Selenosemicarbazone: Cytotoxic Activity Evaluation of Cd(II), Zn(II), Ni(II), Pt(II) and Pd(II) Complexes with Heteroaromatic Selenosemicarbazones. Eur. J. Med. Chem..

[29] Mašković J.M., Hatzidimitriou A., Damjanović A., Stanojković T.P., Trifunović S.R., Geronikaki A.A., Papagiannopoulou D. (2018). MedChemComm Synthesis, Characterization and Biological Evaluation of Pd(II), Cu(II), Re(I) and 99mTc(I) Thiazole-Based Complexes. Med. Chem. Commun..

[30] Malešević N., Srdić T., Radulović S., Sladić D., Radulović V., Brčeski I., Andelković K. (2006). Synthesis and Characterization of a Novel Pd(II) Complex with the Condensation Product of 2-(Diphenylphosphino)Benzaldehyde and Ethyl Hydrazinoacetate. Cytotoxic Activity of the Synthesized Complex and Related Pd(II) and Pt(II) Complexes. J. Inorg. Biochem..

[31] Zafar M.N., Butt A.M., Chaudhry G.e.S., Perveen F., Nazar M.F., Masood S., Dalebrook A.F., Mughal E.U., Sumrra S.H., Sung Y.Y. (2021). Pd(II) Complexes with Chelating N-(1-Alkylpyridin-4(1H)-Ylidene)Amide (PYA) Ligands: Synthesis, Characterization and Evaluation of Anticancer Activity. J. Inorg. Biochem..

[32] Omondi R.O., Bellam R., Ojwach S.O., Jaganyi D., Fatokun A.A. (2020). Palladium(II) Complexes of Tridentate Bis(Benzazole) Ligands: Structural, Substitution Kinetics, DNA Interactions and Cytotoxicity Studies. J. Inorg. Biochem..

