# Supplementary material for: Cytotoxic Effects of New Palladium(II) Complexes with Thiazine or Thiazoline Derivative Ligands in Tumor Cell Lines
Source: Pharmaceutics. 2023 Feb 18;15(2):696. doi: 10.3390/pharmaceutics15020696 (PMC9963275; doi:10.3390/pharmaceutics15020696)
Supplement: Supplementary file 1 [file pharmaceutics-15-00696-s001.zip › pharmaceutics-2164603-supplementary.pdf]

Supplementary Material

Cytotoxic Effects of New Palladium(II) Complexes with Thiazine or Thiazoline Derivative Ligands in Tumor Cell Lines

Elena Fernández-Delgado [1], Samuel Estirado [1], Ana B. Rodríguez [1], Francisco Luna-Giles [2], Emilio Viñuelas-Zahínos [2,*], Javier Espino [1,*] and José A. Pariente [1]

[1] Neuroimmunophysiology and Chrononutrition Research Group, Department of Physiology, Faculty of Science, University of Extremadura, 06006 Badajoz, Spain; elenafd@unex.es (E.F.-D.); estirado@unex.es (S.E.); moratino@unex.es (A.B.R.); pariente@unex.es (J.A.P.)

[2] Coordination Chemistry Research Group, Department of Organic and Inorganic Chemistry, Faculty of Science, University of Extremadura, 06006 Badajoz, Spain; pacoluna@unex.es

* Correspondence: emilvin@unex.es (E.V.-Z.); jespino@unex.es (J.E.); Tel.: +34-924286854 (E.V.-Z.); +34-924289796 (J.E.)

Table S1. Dihedral angles (º) between the planes Cl(1)-Pd-Cl(2) and N(1)-Pd-N(3) in PdPzTn, PdPzTz, PdDMPzTn, PdDMPzTz PdDPhPzTn and PdDPhPzTz complexes

	**PdPzTn**	**PdPzTz**	**PdDMPzTn**	**PdDMPzTz**	**PdDPhPzTn**	**PdDPhPzTz**
Dihedral angle between Cl(1)-Pd-Cl(2) and N(1)-Pd-N(3)	0.0	4.13	2.61	4.65	5.68	7.69

Table S2. Torsion angles (º) for PdPzTn, PdPzTz, PdDMPzTn, PdDMPzTz PdDPhPzTn and PdDPhPzTz complexes and their respective ligands

	**PdPzTn**	**PdPzTz**	**PdDMPzTn**	**PdDMPzTz**	**PdDPhPzTn**	**PdDPhPzTz**
S-C(1)-N(2)-N(3)	- 180.0	174.9(1)	179.3(6)	173.1(2)	- 164.8(1)	158.7(1)
	**PzTn**	**PzTz**	**DMPzTn***	**DMPzTz***	**DPhPzTn**	**DPhPzTz**
	-4.7(2)	-17.9(2)	-	-	0.6(2)	-50.7(2)

*DMPzTn and DMPzTz are oils, so X-ray diffraction data are not available.

Table S3. IR spectral assignments (cm^{-1}) for PzTn, PdPzTn, DMPzTn, PdDMPzTn, DPhPzTn, and PdDPhPzTn.

	**PzTn**	**PdPzTn**	**DMPzTn**	**PdDMPzTn**	**DPhPzTn**	**PdDPhPzTn**
$W_1[\nu(C{=}N)]$	1641	1608	1635	1601	1639	1587
Pyrazole ring vibrations	1514	1532	1574	1571	1560	1556
	1382	1413	1410	1413	1408	1411
	1350	1371	1387	1397	1319	1314
				1378		
	991	1001	970	973	1000	996

Table S4. IR spectral assignments (cm^{-1}) for PzTz, PdPzTz, DMPzTz, PdDMPzTz, DPhPzTz, and PdDPhPzTz.

	**PzTz**	**PdPzTz**	**DMPzTz**	**PdDMPzTz**	**DPhPzTz**	**PdDPhPzTz**
$\Psi_1[\nu(C{=}N)]$	1635	1596	1639	1592	1639	1606
Pyrazole ring vibrations	1510	1524	1566	1564	1548	1556
	1419	1441	1411	1404	1406	1404
	1386	1413	1375	1382		
	1327	1358	1315	1341	1303	1313
	995	1003	981	991	998	1000

Table S5. ^{1}H NMR spectral data for PzTn, PdPzTn, DPhPzTn and PdDPhPzTn complexes in DMF-d_7 solvent and for DMPzTn and PdDMPzTn in DMSO-d_6.

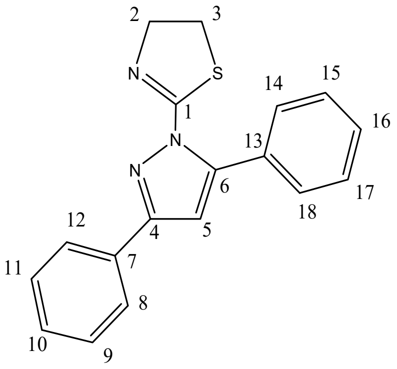

Compound	$N{-}CH_2$	$S{-}CH_2$	H(4)	H(5)	H(6)	CH_3	H(8-18)
PzTn	4.34	3.58	8.40	6.60	7.81	-	-
PdPzTn	4.30	4.13	8.91	6.99	8.15	-	-
DMPzTn	4.27	3.35	-	6.12	-	2.13 ; 2.46	-
PdDMPzTn	4.22	3.98	-	6.60	-	2.63 , 2.65	-
DPhPzTn	4.19	3.51	-	7.20	-		7.46-8.08
PdDPhPzTn	4.22	3.78	-	7.12	-		7.47-7.84

Table S6. ^{1}H NMR spectral data for PzTz, PdPzTz, DPhPzTz and PdDPhPzTz complexes in DMF-d_7.

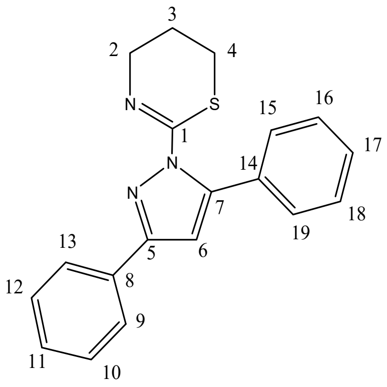

Compound	N-CH_2	H_2(3)	S-CH_2	H(5)	H(6)	H(7)	CH_3	H(9-19)
PzTz	3.83	1.92	3.21	8.33	6.50	7.69	-	-
PdPzTz	3.98	2.22	3.58	8.81	6.92	8.14	-	-
DPhPzTz	3.65	1.91	3.31	-	7.16	-	-	7.47-8.01
PdDPhPzTz	4.02	2.09	3.28	-	7.07	-	-	7.47-7.86

Table S7. Aromatic interactions in PdPzTz, PdDMPzTn, PdDMPzTz, PdDPhPzTn and PdDPhPzTz.

**Compound**	**Type of interaction**	**ANG**	**DC**	**DZ**	**DS**	**ANGS**
PdPzTz	C··H (pyrazole rings)	0	4.887	4.454	4.446	113.10
PdDMPzTn	π -π stacking (pyrazole rings)	1.54	4.340	3.371	4.391	74.67
		1.54	4.452	3.432	4.628	71.87
PdDMPzTz	π -π stacking (pyrazole rings)	1.28	3.916	3.494	3.813	76.80
PdDPhPzTn	T type (phenyl rings)	75.72	4.956	4.908	2.965	137.86
PdDPhPzTz	T type (phenyl rings)	88.08	5.327	5.182	3.156	150.96

Table S8. Cytotoxicity (IC_{50} ± SD, µM) of the different Pd(II) complexes in HeLa cells after 48 and 72h.

	**48h**	**72h**
**PdPzTn**	58.22 ± 9.66 [a]	44.17 ± 6.33 [a]
**PdPzTz**	66.01 ± 11.43 [a]	46.59 ± 6.55 [a]
**PdDMPzTn**	>150 [b]	>150 [b]
**PdDMPzTz**	>150 [c]	>150 [c]
**PdDPhPzTn**	34.85 ± 4.00 [a]	23.28 ± 2.44 [a]
**PdDPhPzTz**	32.09 ± 4.23 [a]	27.98 ± 3.33 [a]

Within each column, values followed by a diverse lowercase letter are significantly different ($P < 0.05$; Tukey's test).

<table>
<tr><th colspan="2">PzTz</th></tr>
<tr><td colspan="2">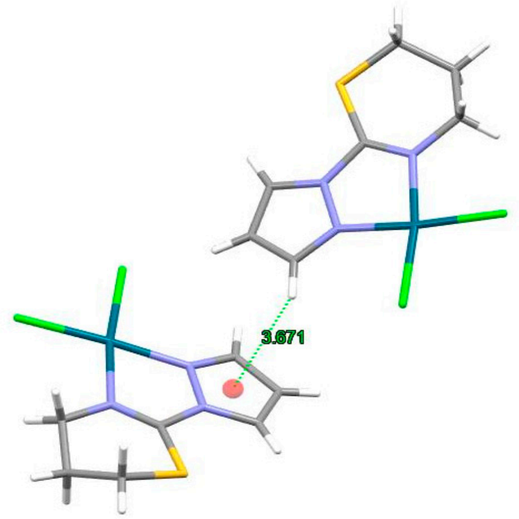
</td></tr>
<tr><th>PdDMPzTn</th><th>PdDMPzTz</th></tr>
<tr><td>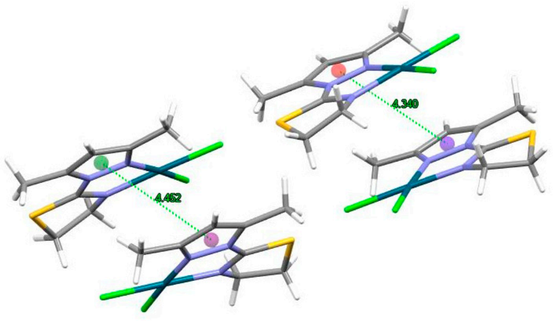</td><td>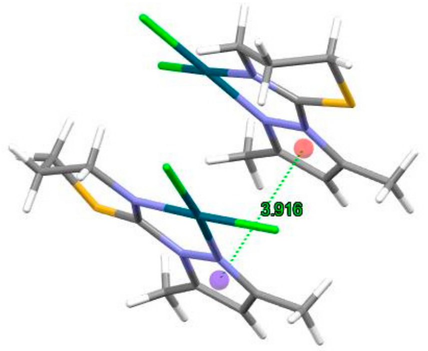
</td></tr>
<tr><th>PdDPhPzTn</th><th>PdDPhPzTz</th></tr>
<tr><td>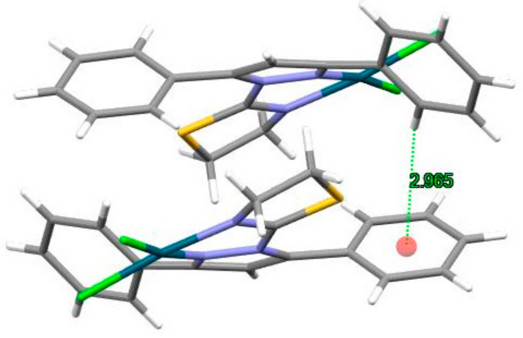
</td><td>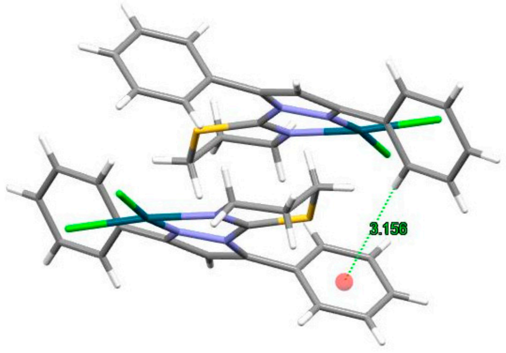
</td></tr>
</table>

Figure S1. Aromatic interactions in PdPzTz, PdDMPzTn, PdDMPzTz, PdDPhPzTn and PdDPhPzTz.

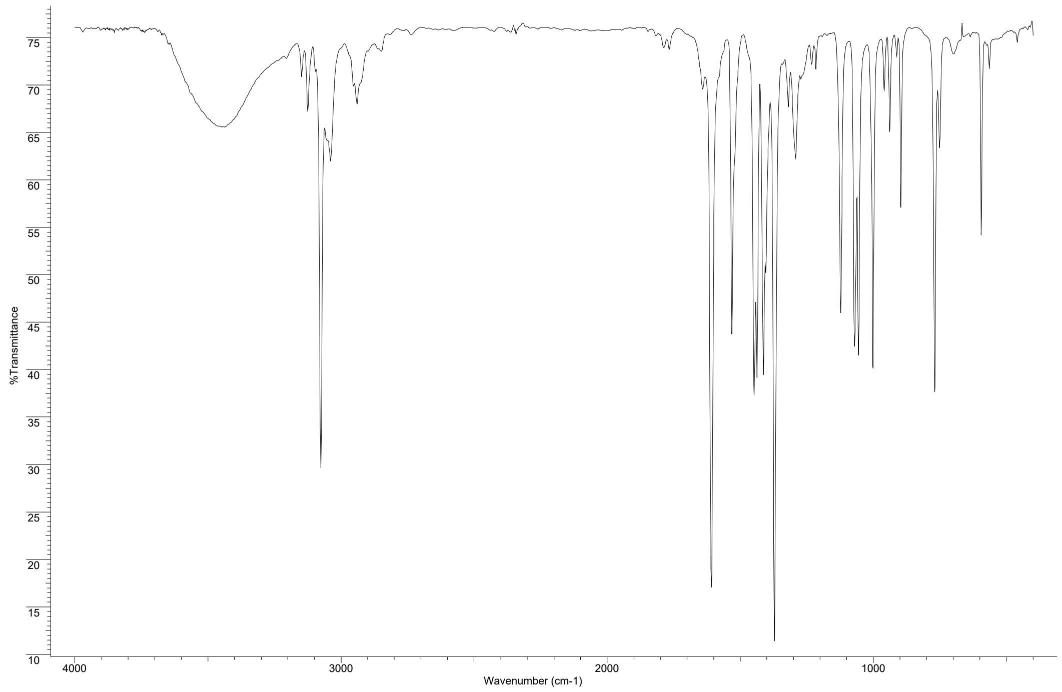

Figure S2. IR spectrum of [$PdCl_2$(PzTn)] in 4000-400 cm^{-1} region.

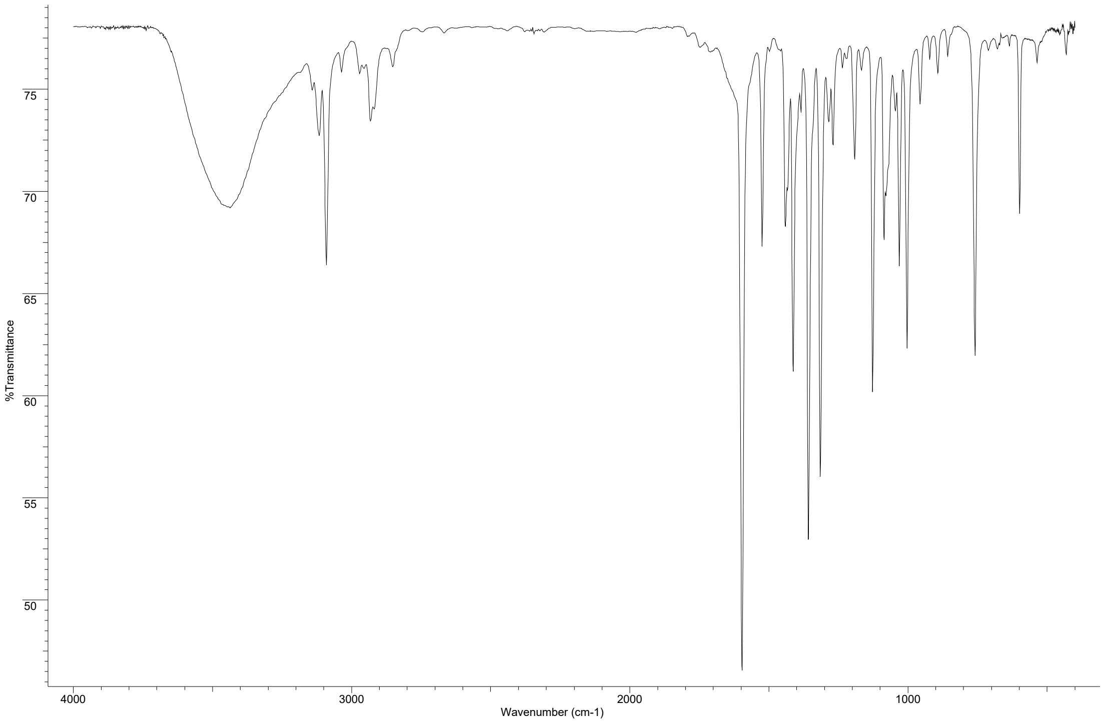

Figure S3. IR spectrum of [$PdCl_2$(PzTz)] in 4000-400 cm^{-1} region.

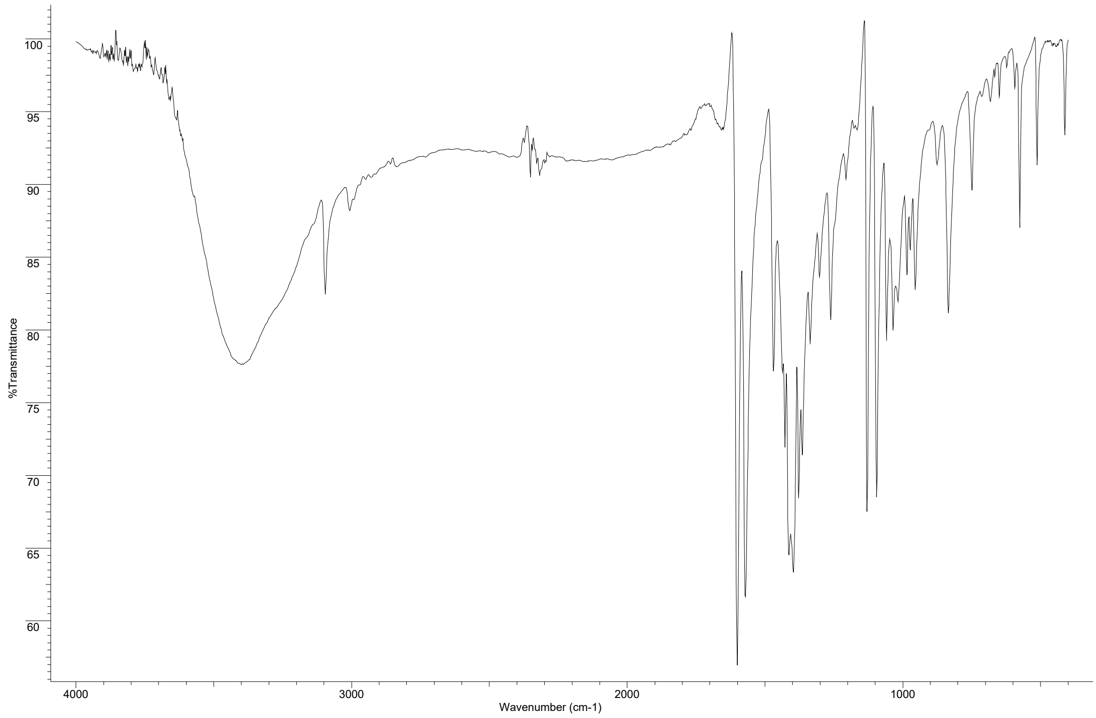

Figure S4. IR spectrum of [$PdCl_2$(DMPzTn)] in 4000-400 cm^{-1} region.

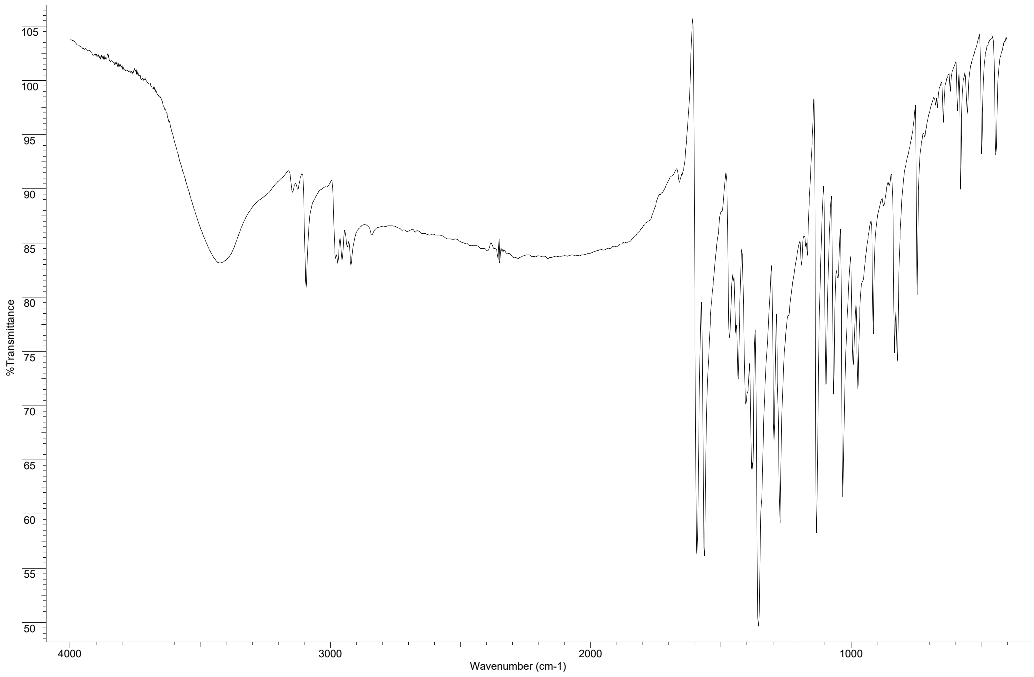

Figure S5. IR spectrum of [$PdCl_2$(DMPzTz)] in 4000-400 cm^{-1} region.

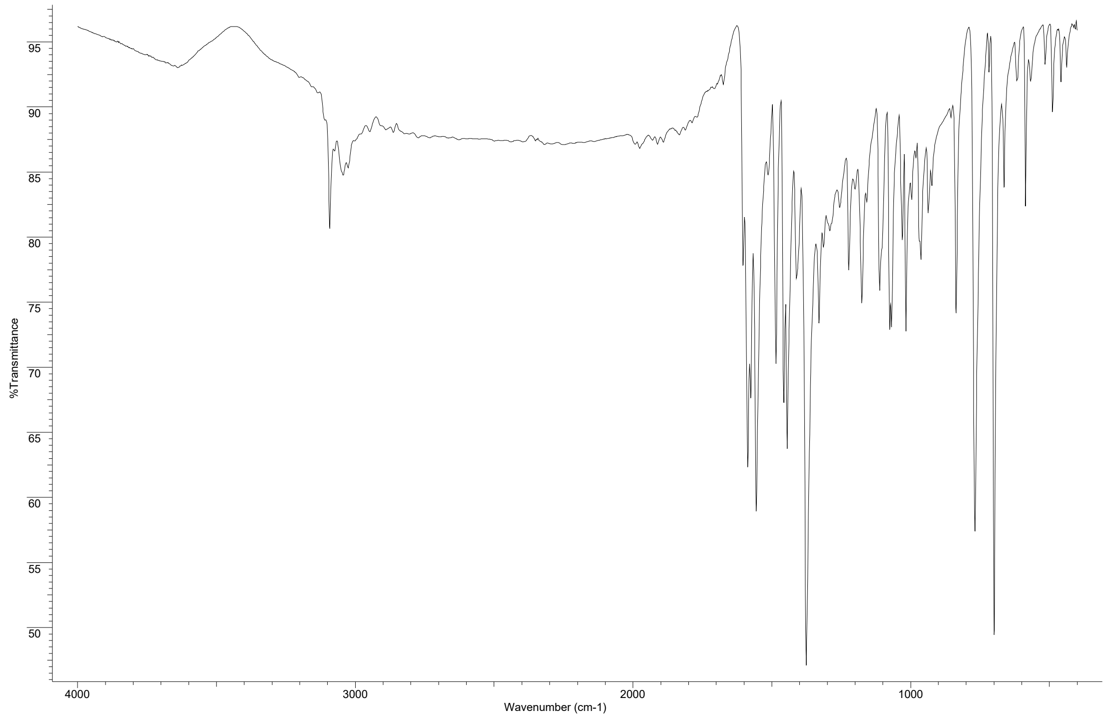

Figure S6. IR spectrum of [$PdCl_2$(DPhPzTn)] in 4000-400 cm^{-1} region.

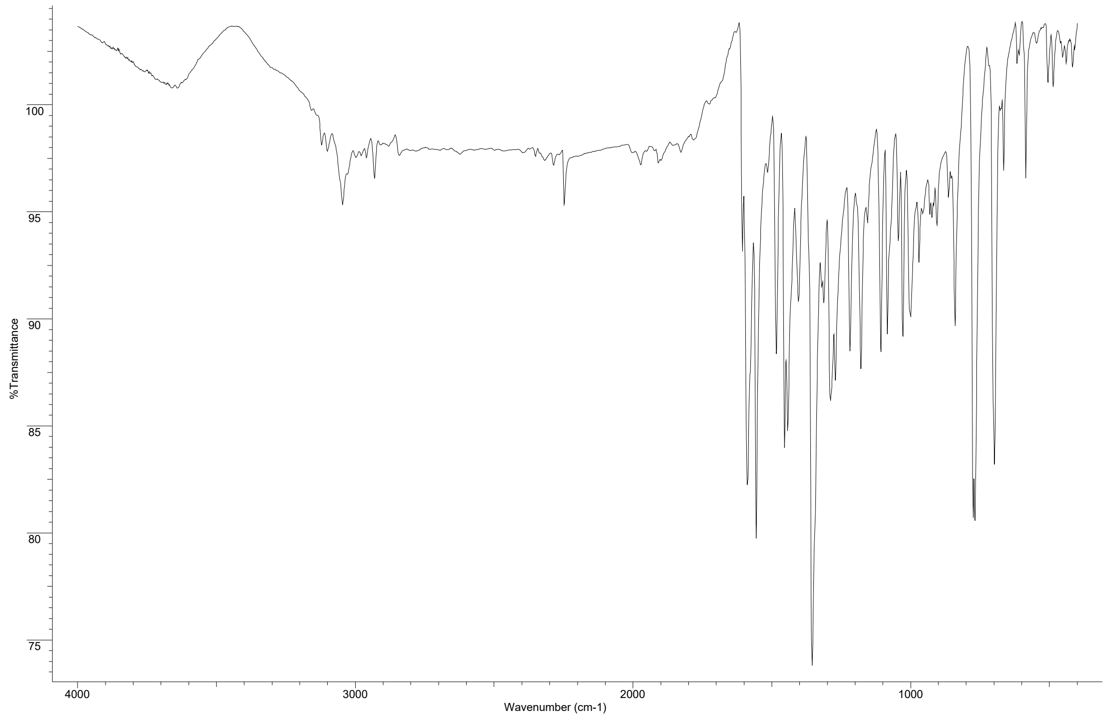

Figure S7. IR spectrum of [$PdCl_2$(DPhPzTz)] in 4000-400 cm^{-1} region.

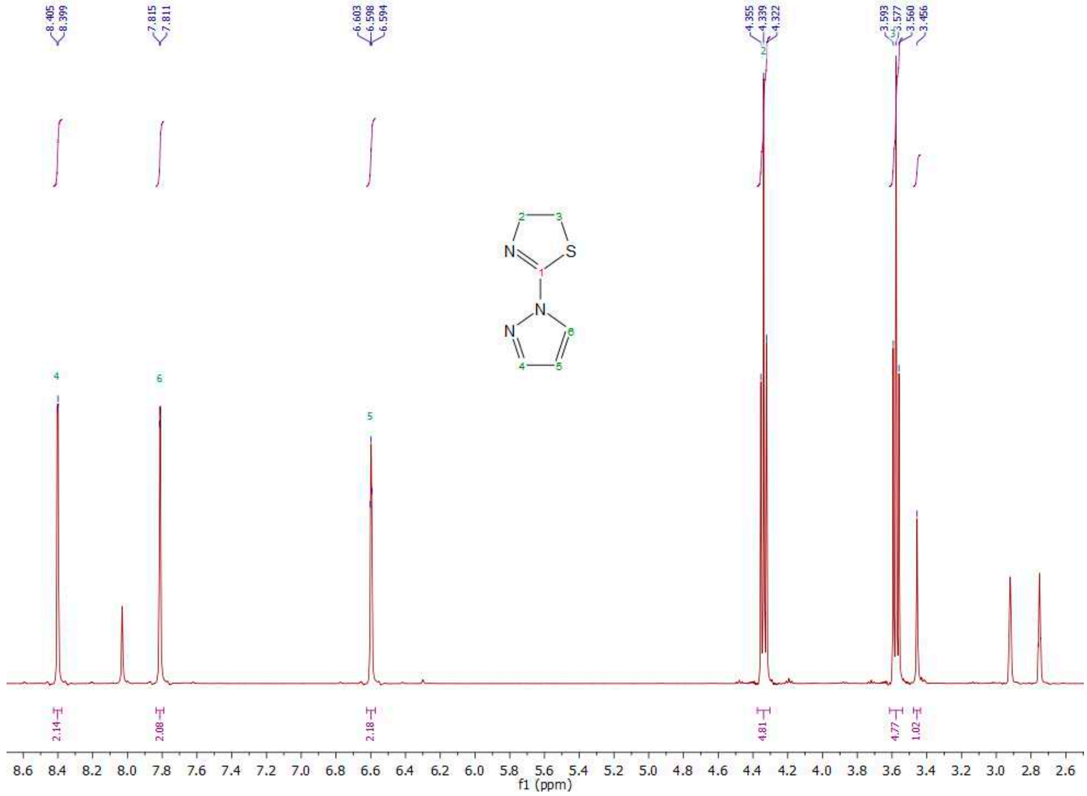

Figure S8. ^{1}H NMR spectrum of PzTn in DMF-d_7.

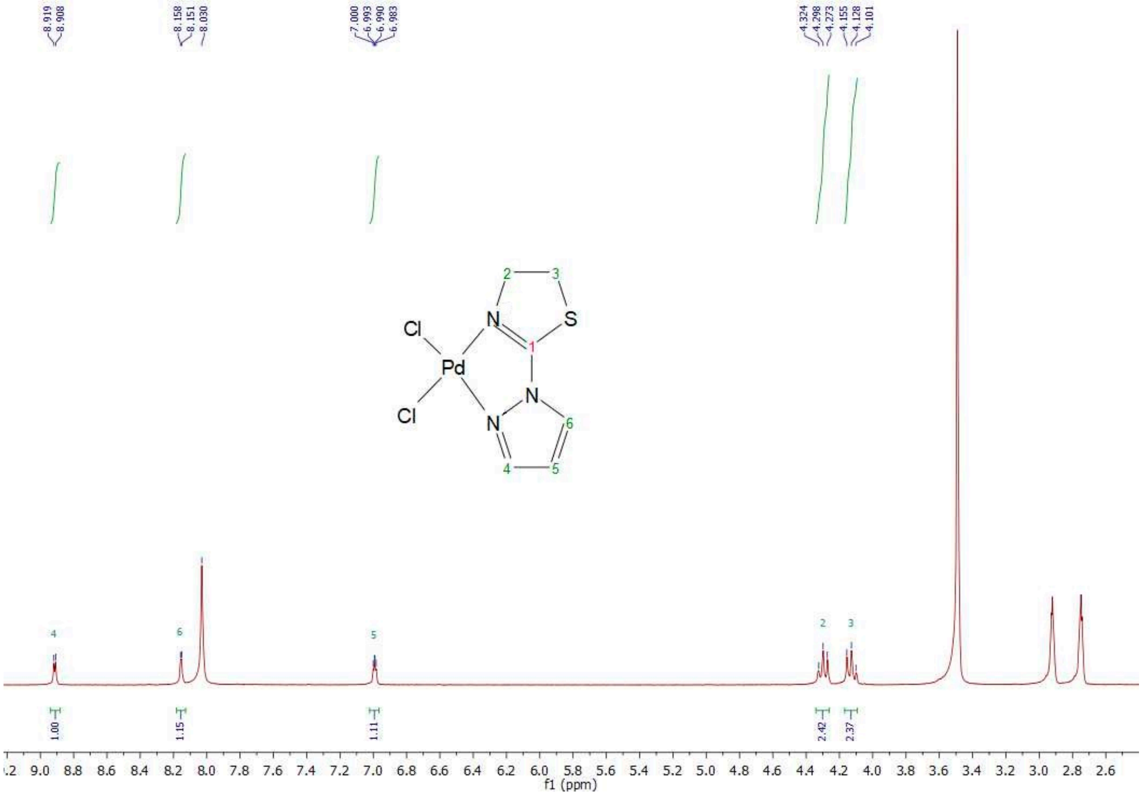

Figure S9. ^{1}H NMR spectrum of PdPzTn in DMF-d_7.

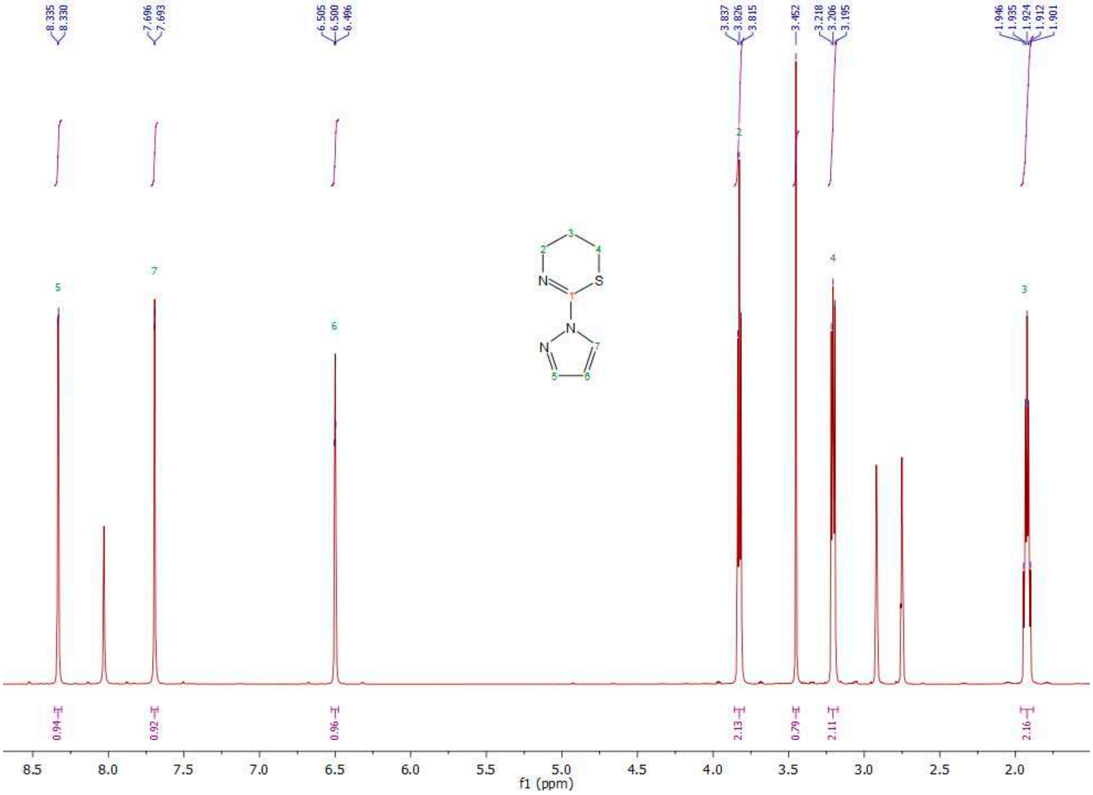

Figure S10. ^{1}H NMR spectrum of PzTz in DMF-d_7.

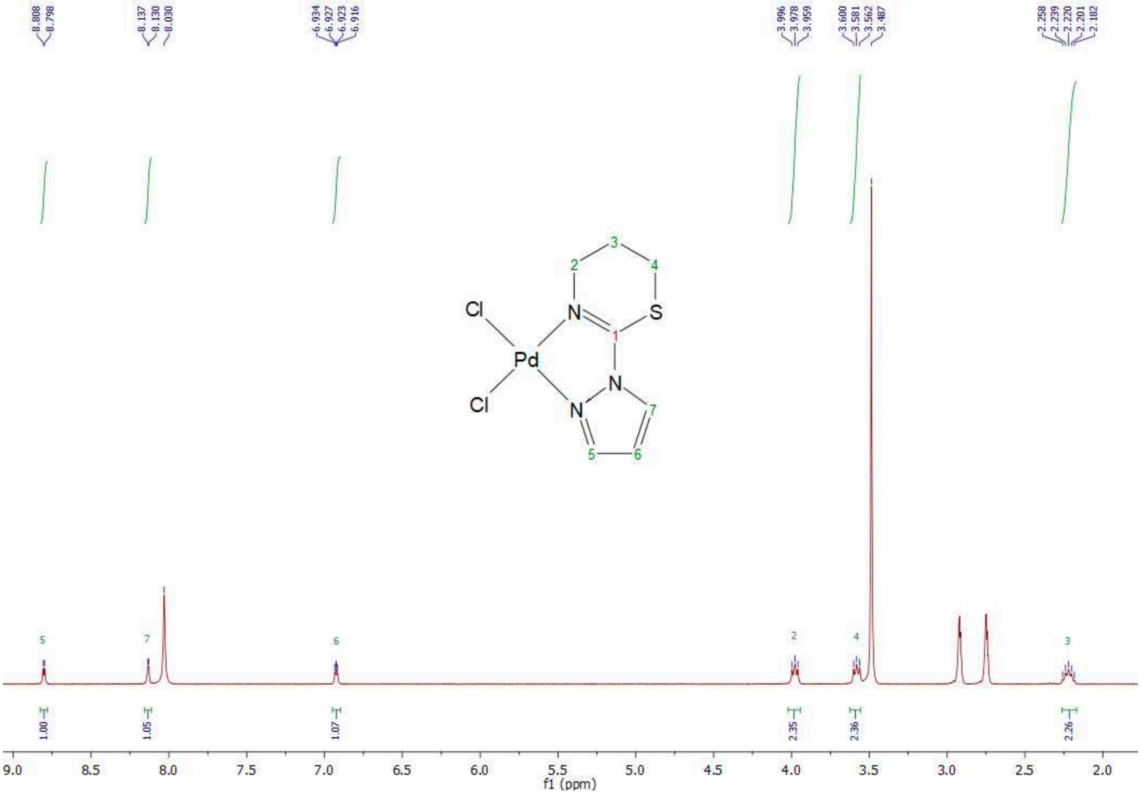

Figure S11. ^{1}H NMR spectrum of PdPzTz in DMF-d_7.

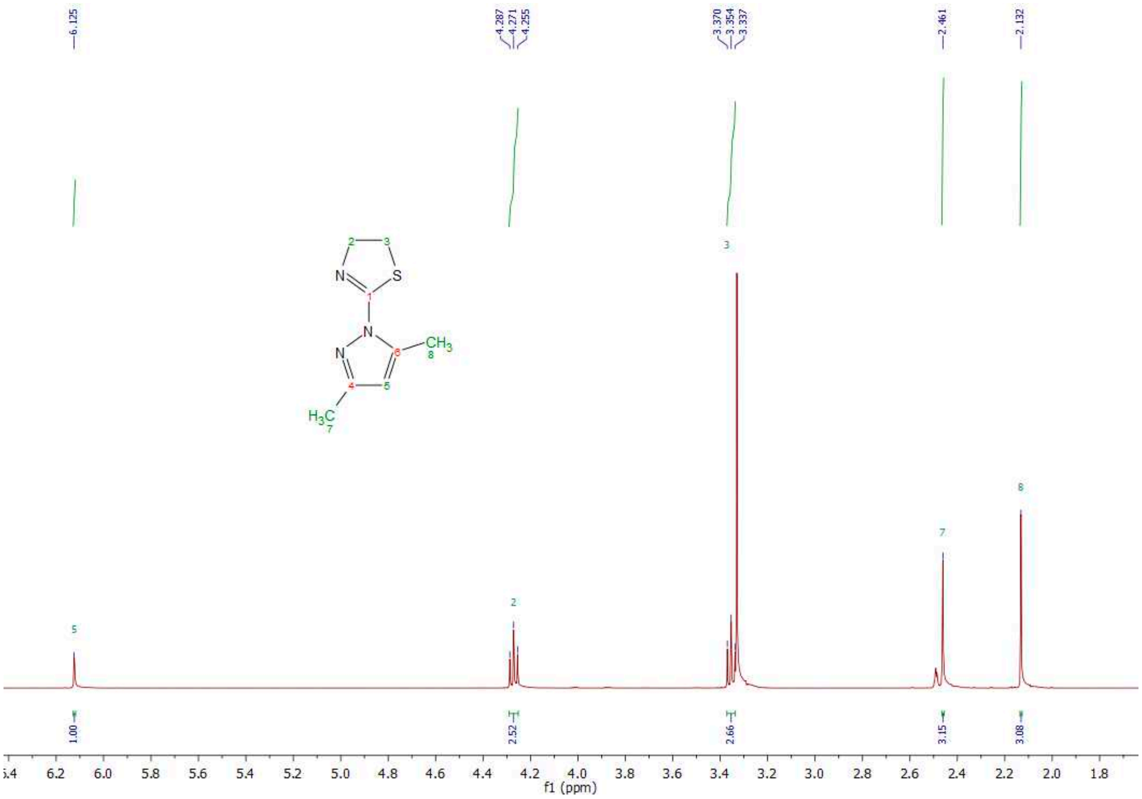

Figure S12. ^{1}H NMR spectrum of DMPzTn in DMSO-d$_6$.

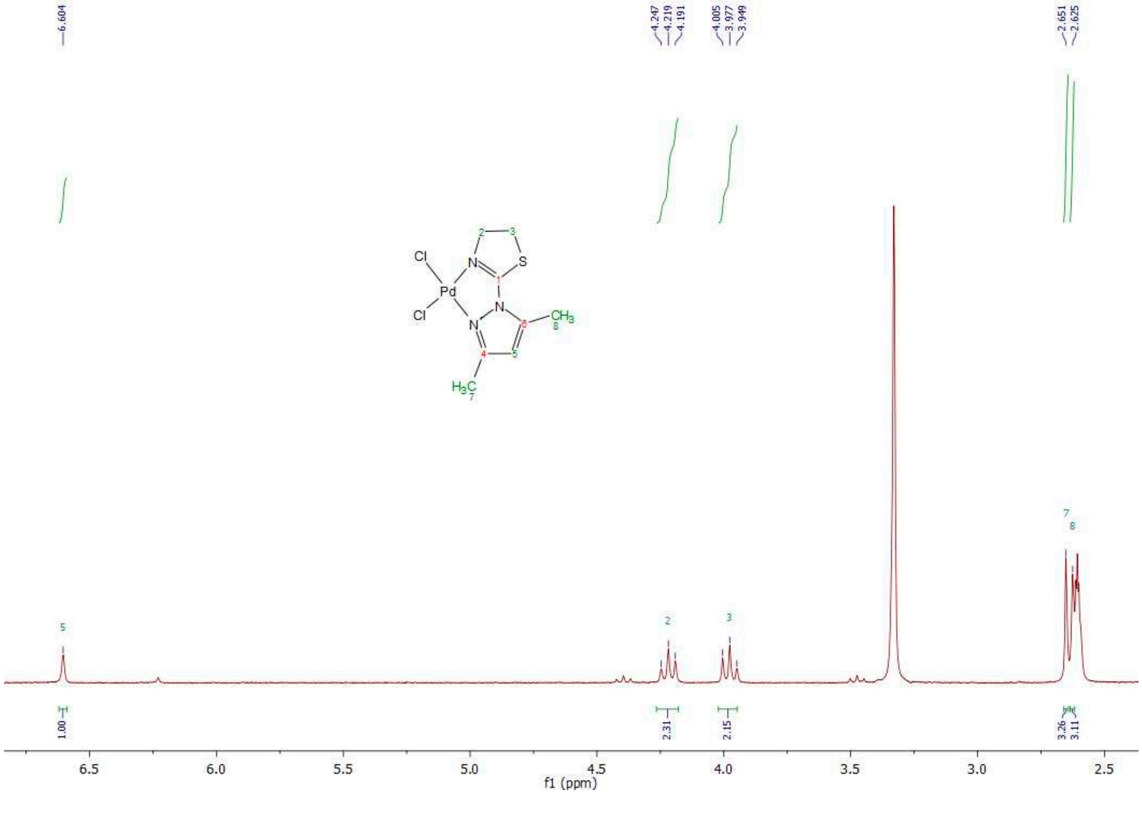

Figure S13. ^{1}H NMR spectrum of PdDMPzTn in DMSO-d$_6$.

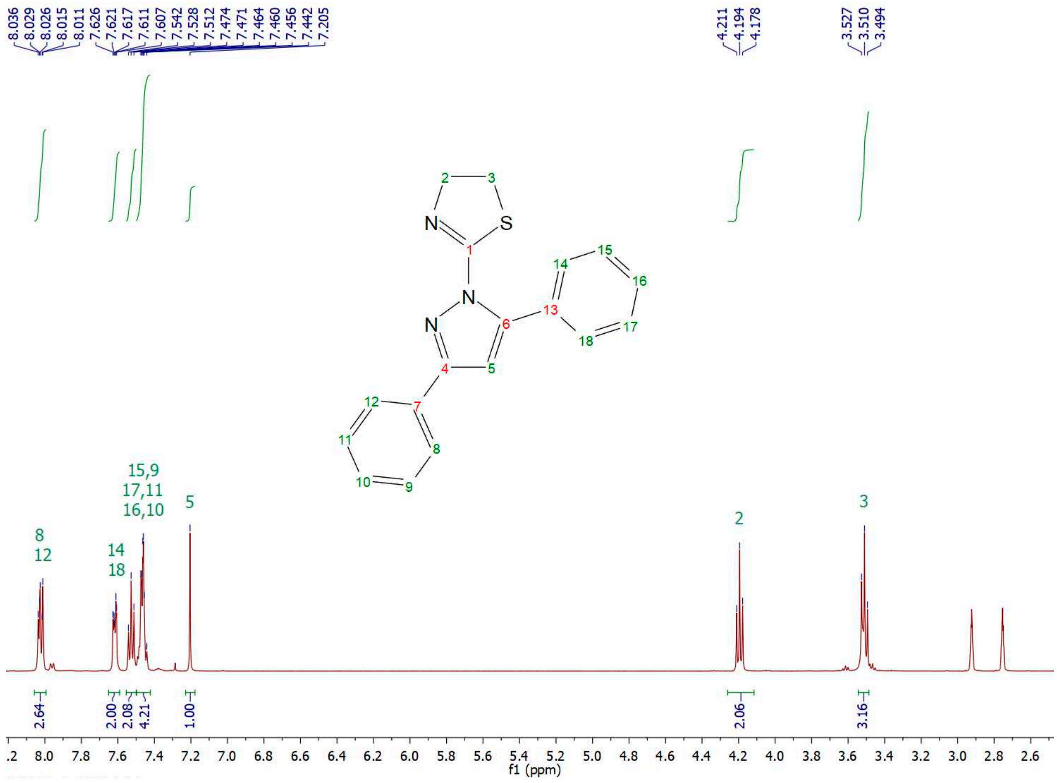

Figure S14. ^{1}H NMR spectrum of DPhPzTn in DMF-d_7.

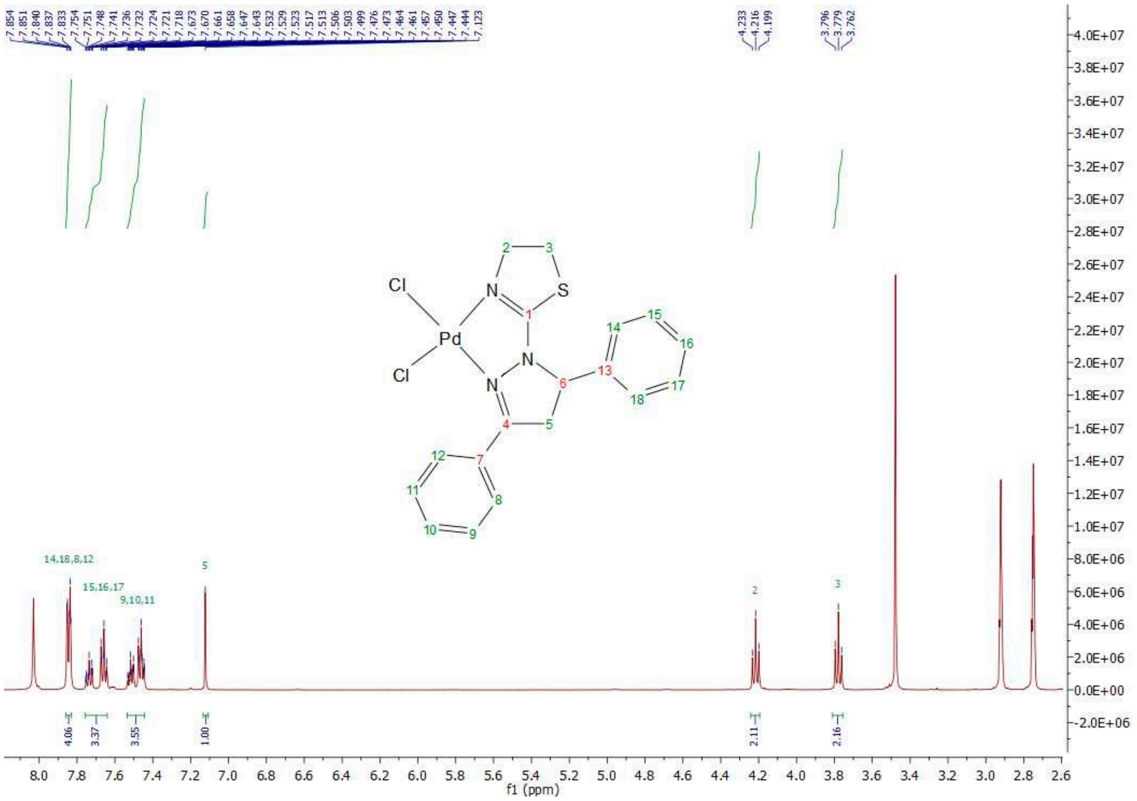

Figure S15. ^{1}H NMR spectrum of PdDPhPzTn in DMF-d_7.

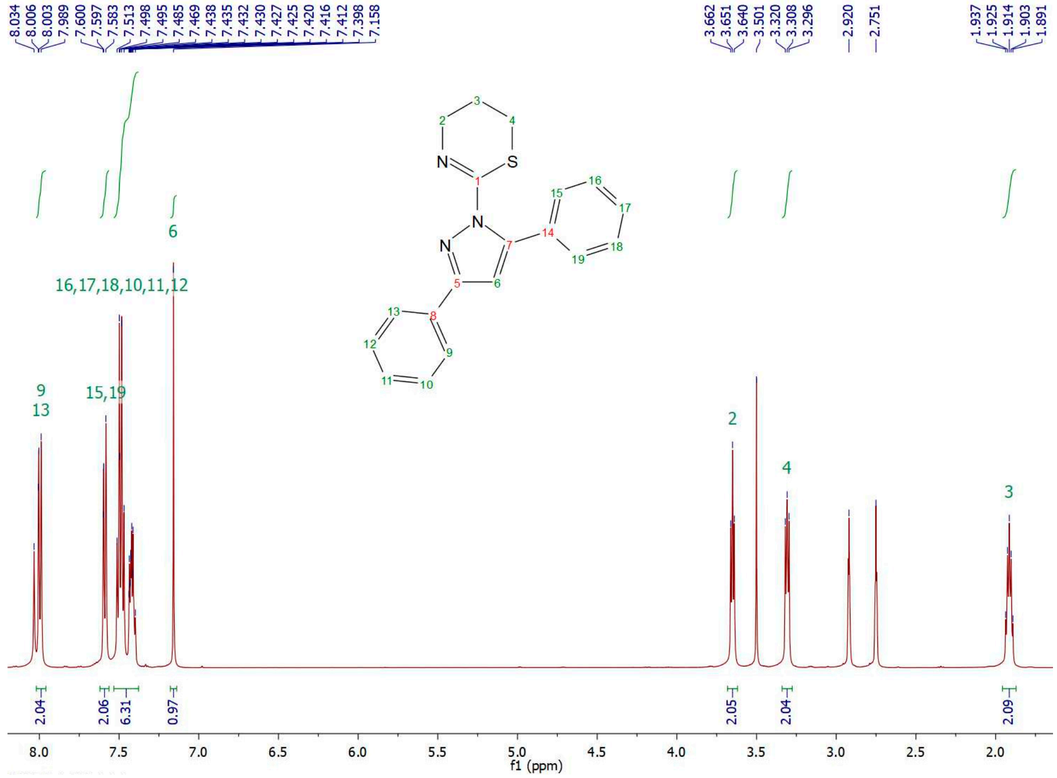

Figure S16. ^{1}H NMR spectrum of DPhPzTz in DMF-d_7.

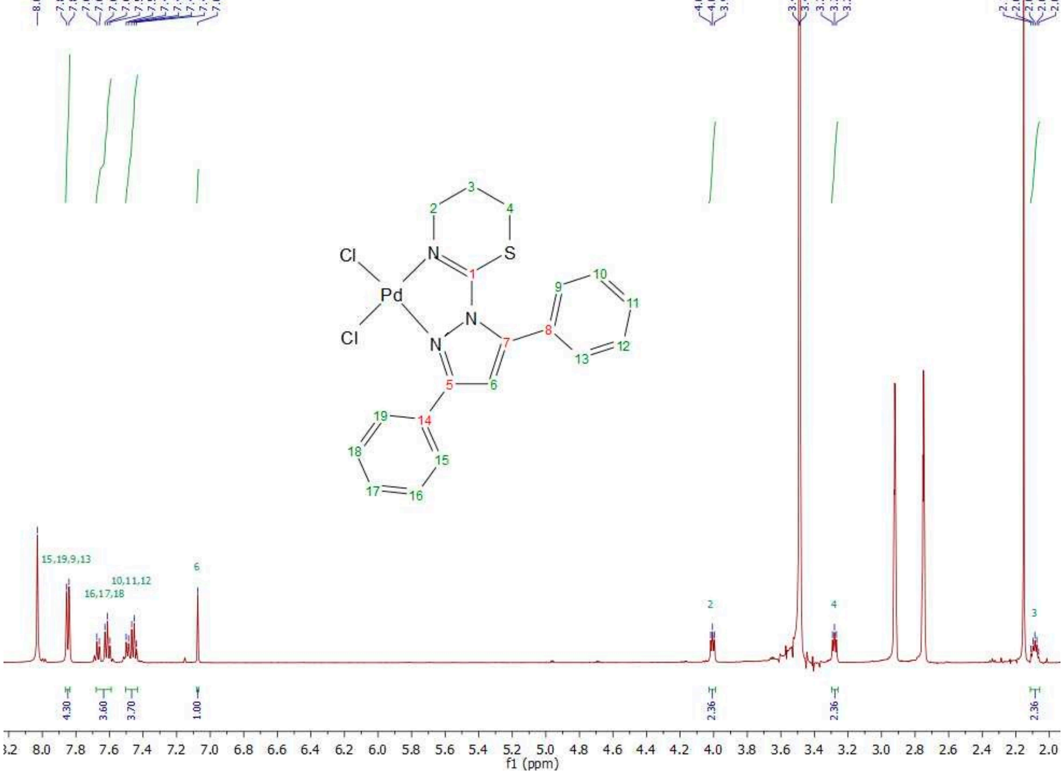

Figure S17. ^{1}H NMR spectrum of PdDPhPzTz in DMF-d_7

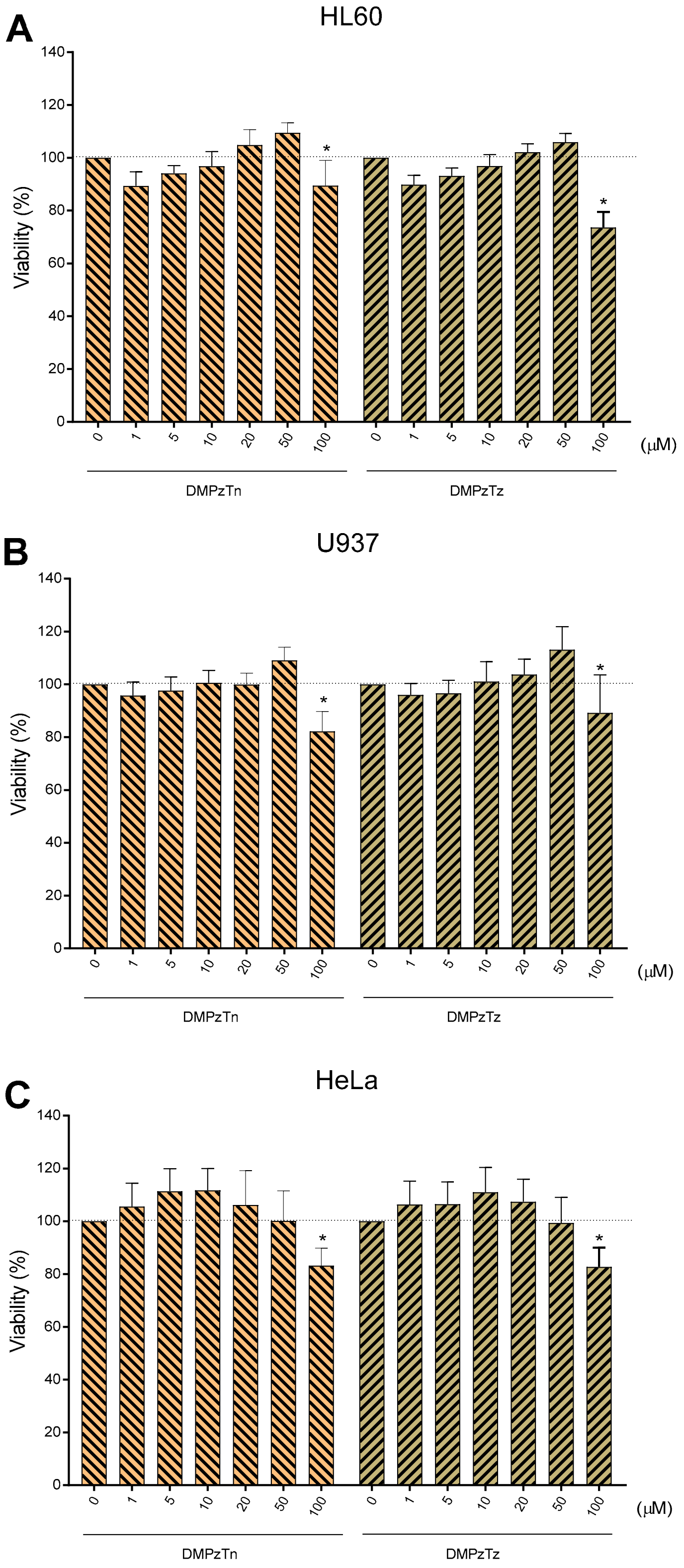

Figure S18. Dose-response curves of the pyrazole/thiazoline ligands on cell viability. HL-60 (A), U-937 (B), and HeLa (C) cells were treated for 24 h with increasing concentrations (0-100 μM, as indicated) of the ligands DMPzTn and DMPzTz, or the vehicle (DMSO, control). Data represent means ± S.D. of 5 independent experiments and are expressed as percentage of control values. *$P < 0.05$ compared to their corresponding control values.

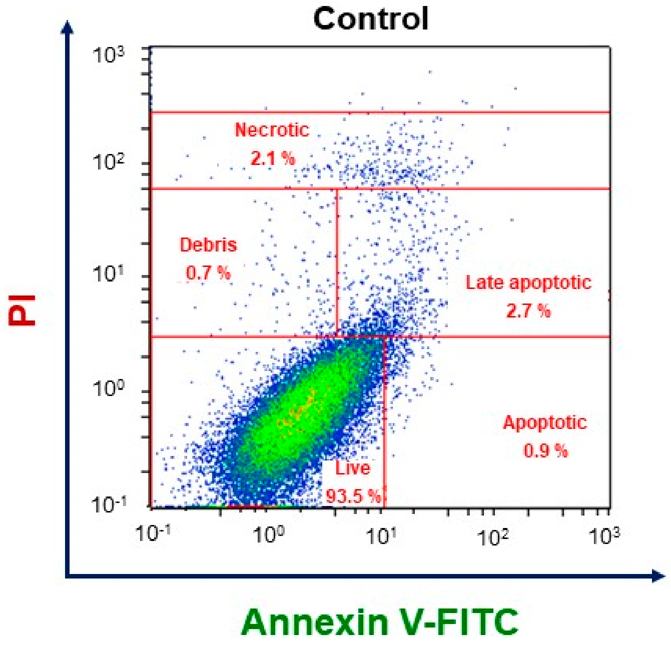

Figure S19. Representative cytogram of untreated HeLa cells stained with annexin V-FITC in the presence of propidium iodide (PI).
